# Phytochemical Investigation of *Rhus typhina* Drupes and Identification of a New Antibacterial Lupane-Type Triterpenoid

**DOI:** 10.3390/molecules31214012

**Published:** 2026-11-06

**Authors:** Marie Frissard, Marianne Piochon, Jean Legault, Vakhtang Mshvildadze, Nassim Djabou, Jérôme Alsarraf, Antoine Desportes, Josianne Savard, André Pichette

**Affiliations:** Laboratoire d’Analyse et de Séparation des Essences Végétales, Centre de Recherche sur la Boréalie, Département des Sciences Fondamentales, Université du Québec à Chicoutimi, Saguenay, QC G7H 2B1, Canada; mfrissar@uqac.ca (M.F.); mpiochon@uqac.ca (M.P.); jlegault@uqac.ca (J.L.); vmshvild@uqac.ca (V.M.); ndjabou@uqac.ca (N.D.); jalsarra@uqac.ca (J.A.); adesport@uqac.ca (A.D.); j3savard@uqac.ca (J.S.)

**Keywords:** boreal forest, *Rhus typhina* L., drupes, antibacterial, triterpene, 3α-hydroxy-3β,25-epoxylup-20(29)-ene

## Abstract

The antibacterial constituents of *Rhus typhina* drupes remain poorly characterized despite their traditional medicinal use to treat infections. The methanolic extract of *R. typhina* drupes was evaluated for its antibacterial activity. It inhibited *Escherichia coli* (IC_50_ = 12 ± 1 µg/mL) and *Staphylococcus aureus* (IC_50_ = 33 ± 8 µg/mL). The extract was subjected to partial bioassay-guided fractionation and phytochemical investigation, leading to the isolation of eighteen compounds whose structures were elucidated by mass spectrometry and nuclear magnetic resonance spectroscopy. A new lupane-type triterpenoid, 3α-hydroxy-3β,25-epoxylup-20(29)-ene (**1**), was identified and characterized by high-resolution mass spectrometry, IR spectroscopy, and detailed 1D and 2D NMR analyses. HMBC and NOESY correlations supported a lupane-type skeleton bearing a ring-A hemiketal functionality and established its relative stereochemistry. Antibacterial activity was assessed against *E. coli* and *S. aureus* for the six isolated compounds available in sufficient quantities for testing. Compound **1** showed activity against *S. aureus* (IC_50_ of 0.052 ± 0.004 µM), while its cytotoxicity IC_50_ toward human WS-1 fibroblasts was >100 µM. Hyperoside (**16**) was also active against *S. aureus* (IC_50_ of 0.48 ± 0.09 µM). These results identify compound **1** as a candidate for further investigation of its antibacterial properties.

## 1. Introduction

Natural products have historically played a central role in anti-infective drug discovery [1]. Plants, in particular, produce numerous secondary metabolites that contribute to defense against microbial pathogens [2]. Indigenous populations of North America have traditionally used more than 2600 plant species for medicinal purposes, selecting them based on empirically observed therapeutic effects [3,4]. Many modern drugs are derived from plants historically used in traditional medicine. For example, paclitaxel (Taxol), a taxane diterpenoid widely used as an anticancer agent, was originally isolated from the bark of the Pacific yew, a tree used in Native American ethnomedicine [5,6]. Recent advances in analytical chemistry have expanded our ability to explore plant metabolism, thereby accelerating the discovery of plant-derived compounds with beneficial properties [7].

Boreal forest plants represent a valuable source of bioactive compounds, and their chemical diversity and biological potential continue to be explored. These plants grow under harsh environmental conditions, which often induce the production of structurally diverse secondary metabolites such as terpenoids, phenolics, flavonoids, and alkaloids. Many of these compounds possess antimicrobial properties, contributing to the plant’s defense against bacterial and other pathogen attacks [8,9]. Among the boreal flora, *Rhus typhina* L. (staghorn sumac or vinegar tree) is of particular interest for its reported biological properties [10]. This shrub is a member of the *Anacardiaceae* family native to North America and belongs to the genus *Rhus*, which comprises more than 250 species [11]. Botanically, it is a small tree with pinnately compound leaves and young stems densely covered with velvety, reddish-brown hairs resembling the velvet of a stag’s antlers. It produces dense, terminal, cone-like clusters of flowers that mature into the characteristic bright red, fuzzy drupes. According to historical ethnobotanical records, these fruits were traditionally utilized by various Native American tribes to prepare remedies for gastrointestinal ailments, such as diarrhea and vomiting, as well as for localized infections including sore throats and venereal conditions [12,13,14,15,16]. Beyond their medicinal uses, *R. typhina* fruits were also valued as a source of food, beverages, and natural dyes [17]. Today, they continue to be used in contemporary applications, including perfumery and as a natural preservative [10]. Ethanolic extracts of the drupes display antimicrobial activity against *Pseudomonas aeruginosa*, *Escherichia coli*, and *Staphylococcus aureus* [18], likely linked to their high content of gallotannins [19]. Polyphenols are among the most abundant secondary metabolites reported in the genus *Rhus*, including phenolic acids (gallic acid, hydroxybenzoic and hydroxycinnamic acids) and flavonoids such as anthocyanins and quercetin-3-*O*-rhamnoside [20,21]. In addition to these compounds, several *Rhus* species have been reported to contain other classes of secondary metabolites, including tannins, lignans, coumarins, and triterpenoids. Notably, *R. chinensis* [22,23] and *R. coriaria* [24,25] have been extensively studied and shown to possess a rich phytochemical diversity. In contrast, despite its documented ethnomedicinal uses and antibacterial activity, the phytochemical composition of *R. typhina* remains comparatively underexplored.

Continuing our research on antibacterial compounds derived from boreal forest plants [26,27], the present study focuses on evaluating the biological activity and isolating the active constituents of *R. typhina* drupes. We hypothesized that the antibacterial activity previously reported for the drupes is associated with specific secondary metabolites that can be isolated and characterized through phytochemical investigation, supported by preliminary antibacterial screening, a strategy that has historically contributed to the discovery of numerous therapeutic agents [28,29]. Accordingly, the objectives of this study were to characterize the methanolic extract of *R. typhina* drupes through phytochemical and biological screening and to isolate and identify antibacterial constituents using bioassay-guided fractionation. In total, eighteen compounds were identified, including a new lupane-type triterpenoid.

## 2. Results and Discussion

### 2.1. Phytochemical Investigation and Bioassay-Guided Fractionation

The dried drupes of *R. typhina* were extracted with methanol (MeOH) by ultrasonication. This solvent was selected because it efficiently extracts a broad range of secondary metabolites spanning a wide range of polarities. This procedure yielded a sticky red crude extract (24.7%, *w*/*w*) of the initial dry mass of the drupes. As shown in Table 1, the extract showed antibacterial activity against *E. coli* (IC_50_ = 12 ± 1 µg/mL) and *S. aureus* (IC_50_ = 33 ± 8 µg/mL). A portion of the crude methanolic extract was then subjected to liquid–liquid partitioning between water and ethyl acetate (EtOAc) to remove highly polar constituents, particularly sugars. Based on the mass of extract subjected to partitioning, the dried aqueous and EtOAc fractions represented 46.2% and 52.4% respectively, with a mass loss of 1.3%. The aqueous fraction showed only weak antibacterial activity (IC_50_ = 127 ± 8 µg/mL against *E. coli* and >200 µg/mL against *S. aureus*), consistent with the enrichment of highly polar constituents during liquid–liquid partitioning. In contrast, the EtOAc fraction retained strong antibacterial activity, with IC_50_ values of 7.7 ± 0.9 µg/mL against *E. coli* and 31 ± 8 µg/mL against *S. aureus* (Table 1), supporting its selection for further fractionation.

Fractionation of a portion of the EtOAc fraction on Diaion HP-20 resin using a stepwise gradient of MeOH in water afforded four subfractions (A–D). The results of the preliminary antibacterial screening, which guided subsequent fractionation, are summarized in Table 1, while their thin-layer chromatographic (TLC) profiles are presented in Figure S1 (Supplementary Materials). The tentative assignment of compound classes was based on the overall TLC profiles, considering the migration behavior and the visualization patterns of the chromatographic bands under ultraviolet (UV) light and after chemical derivatization. Fraction A (31.3%) showed a TLC profile dominated by blue to blue-green fluorescent bands after NP/PEG derivatization under UV light at 365 nm, suggesting an enrichment in phenolic acids. It exhibited IC_50_ values of 22 ± 1 µg/mL against *E. coli* and 29 ± 5 µg/mL against *S. aureus*. Fraction B (22.2%) showed a distinct TLC profile characterized predominantly by yellow-to-orange fluorescent bands after NP/PEG derivatization, suggesting an enrichment in flavonoid-type phenolic constituents. This fraction also exhibited antibacterial activity, with IC_50_ values of 5.6 ± 0.6 µg/mL against *E. coli* and 33 ± 6 µg/mL against *S. aureus*. Fraction C (32.8%) exhibited a TLC profile that substantially overlapped with those of fractions B and D. It showed IC_50_ values of 7 ± 1 µg/mL against *E. coli* and 22 ± 9 µg/mL against *S. aureus*. Fraction D (12.4%), in contrast, exhibited a TLC profile dominated by less strongly retained constituents, suggesting an enrichment in less polar compounds. Initially, fraction D appeared inactive (IC_50_ > 200 µg/mL for both strains), possibly due to its insolubility in the aqueous culture medium. To overcome this limitation, fraction D was dissolved in DMSO prior to addition to the culture medium, while maintaining the final DMSO concentration below 0.5%. Under these conditions, fraction D exhibited antibacterial activity, with IC_50_ values of 26 ± 6 µg/mL against *E. coli* and 25 ± 2 µg/mL against *S. aureus*. These results suggest that hydrophobic constituents present in fraction D contribute to the antibacterial activity observed after solubilization.

Although fraction C was the largest subfraction, its TLC profile substantially overlapped with those of fractions B and D, and its antibacterial activity against *E. coli* and S. *aureus* was comparable to that of these fractions. Fraction C was therefore not subjected to further fractionation, as additional purification was considered likely to result predominantly in the re-isolation of constituents already represented in fractions B and D. This approach prioritized fractions with distinct chromatographic profiles and greater chemical diversity while minimizing redundant isolation efforts. However, it may have resulted in the omission of constituents unique to fraction C. The isolation procedure was therefore focused on fractions A, B, and D, leading to the isolation of one new lupane-type triterpenoid (**1**) and seventeen known compounds (**2**–**18**).

### 2.2. Structural Elucidation of Compound ***1*** and Identification of Compounds ***2**–**18***

Compound **1** was isolated from fraction D as a pale-yellow crystalline powder. Its molecular formula was determined by high-resolution mass spectrometry to be C_30_H_48_O_2_, based on the pseudo-molecular ion *m*/*z* 441.3710 [M+H]^+^ (Figure S2). The molecular ion at *m*/*z* 441, along with the C_30_ molecular formula, was indicative of a triterpenoid skeleton. Compared with a non-functionalized triterpene (C_30_H_48_; 408 g/mol), the observed mass increase of 32 Da was consistent with the presence of two oxygen atoms [30]. In-source dehydration ions were observed at *m*/*z* 423.3614 ([M+H–H_2_O]^+^) and 405.3511 ([M+H–2H_2_O]^+^) in the mass spectrometry (MS) spectrum, consistent with the presence of hydroxyl functionalities and with patterns previously reported for related pentacyclic triterpenoids. Tandem mass spectrometry (MS/MS) experiments performed on the precursor ion yielded limited fragmentation, consistent with a rigid scaffold bearing few labile functional groups [31].

**Figure 1:**
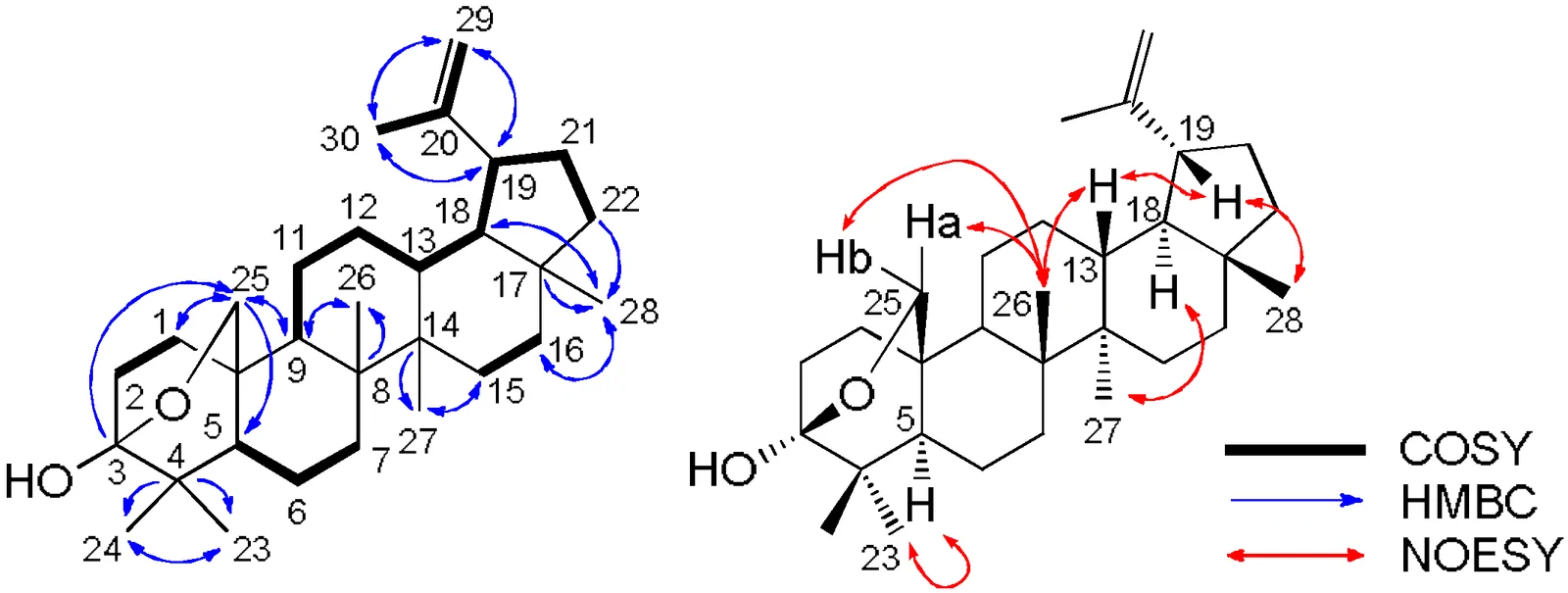
Key ^1^H–^1^H COSY, ^1^H–^13^C HMBC and NOESY correlations used to establish the structure and relative stereochemistry of compound **1**.

The structural assignment was confirmed by detailed analysis of the nuclear magnetic resonance (NMR) spectra (Table 2 and Table S1, Figures S2–S12). Preliminary analysis of the NMR data suggested a lupane-type triterpenoid, based on characteristic signals for a terminal olefin at δ_H_ 4.68 (d, *J* = 2.4 Hz) and 4.56 (dq, *J* = 2.7, 1.4 Hz), together with signals at δ_C_ 150.9 (C-20) and 109.5 (C-29) [32,33]. The HMBC spectrum further supported the presence of the isopropenyl group through correlations of H-30 (δ_H_ 1.67) with C-20 (δ_C_ 150.9), C-29 (δ_C_ 109.5), and C-19 (δ_C_ 48.0) (Figure 1). The presence of six singlet methyl signals at δ_H_ 1.67, 1.01, 0.96, 0.94, 0.92, and 0.78 further supported the lupane skeleton. The ^13^C NMR and HSQC data confirmed the corresponding methyl carbons at δ_C_ 27.0 (C-23), 19.5 (C-30), 18.5 (C-24), 18.2 (C-28), 16.4 (C-26) and 14.4 (C-27). Two additional downfield proton signals at δ_H_ 4.24 (dd, *J* = 8.8, 2.9 Hz) and 3.72 (dd, *J* = 8.8, 1.9 Hz), together with a ^13^C signal at δ_C_ 68.2 (C-25), were attributed to a hemiketal methylene group. A literature search for triterpenoids from the genus *Rhus* revealed that compound **1** shared several spectral features with lantabetulic acid, particularly the hemiketal function in ring A involving a bridge between C-3 (δ_C_ 98.9) and C-25 (δ_C_ 68.2) [32,34]. This assignment was further supported by an HMBC correlation between C-3 (δ_C_ 98.9) and H-25b (δ_H_ 3.72). The ^1^H–^1^H COSY data showed W-type coupling between H-25b (δ_H_ 3.72) and H-5 (δ_H_ 1.16), and between H-25a (δ_H_ 4.24) and H-1b (δ_H_ 1.10), consistent with the 3β,25-epoxy system described by Schmidt et al. [35]. Although the quaternary carbon C-3 (δ_C_ 98.9) was not directly observed in the ^13^C NMR spectrum, its presence was inferred from HMBC correlations with the methyl protons H-23 (δ_H_ 1.01) and H-24 (δ_H_ 0.96). ^1^H–^1^H COSY correlations between the methine proton H-5 (δ_H_ 1.16) and the methylene protons H-6 (δ_H_ 1.57, 1.45), and between H-6 and H-7 (δ_H_ 1.43, 1.30), supported the C-5–C-6–C-7 sequence within the ring system. Similarly, ^1^H–^1^H COSY correlations between H-9 and H-11, H-11 and H-12, H-12 and H-13, H-13 and H-18, H-18 and H-19, H-19 and H-21, and H-21 and H-22 supported the proton connectivity and the assignment of corresponding carbon positions within the fused ring system. HMBC correlations from H-26 to C-7, C-8, and C-14 confirmed the position of the C-26 methyl group (δ_C_ 16.4). Similarly, correlations from H-27 to C-8, C-13, and C-15, from H-28 to C-22 and C-16, and from H-30 to C-20 and C-19 supported the assignments of the C-27 (δ_C_ 14.4), C-28 (δ_C_ 18.2), and C-30 (δ_C_ 19.5) methyl groups.

The relative stereochemistry was established based on NOESY correlations and the coupling constants of the relevant protons in the ^1^H NMR spectrum (Figure 1). NOESY correlations between H-25a and H-26 (δ_H_ 4.24 and 0.94) and between H-25b and H-26 (δ_H_ 3.72 and 0.94) were consistent with the proposed stereochemical arrangement of the 3β,25-epoxy system. Similarly, NOESY correlations between H-26 and H-13 (δ_H_ 0.94 and 1.66), H-13 and H-19 (δ_H_ 1.66 and 2.36), and H-19 and H-28 (δ_H_ 2.36 and 0.78) supported the β-orientation of the C-28 methyl group and the α-orientation of the isopropenyl group at C-19.

Compound **1** was identified as 3α-hydroxy-3β,25-epoxylup-20(29)-ene. Comparison with published NMR data for structurally related triterpenes further supported the proposed structure, particularly the 3α-hydroxy-3β,25-epoxy system [34,35,36,37], the C-28 methyl group [35,38], and the isopropenyl group linked to C-19 [34,37]. Structure searches in the PubChem and SciFinder databases yielded no records matching this exact structure, which was also absent from a comprehensive phytochemical study of *R. typhina* fruits [21] and reviews of compounds identified in *Rhus* species [21,22,23,39]. Several structurally related triterpenes have been reported in the genus *Rhus*, including lantabetulic acid [37,39,40], lantabetulal [37], methyl lantabetulate [40], 3β,25−dihydroxy-lup-20(29)-ene [41], and 3α,20-dihydroxy-3β,25-epoxylupane which was isolated from the flowers of *Rhus typhina* [35]. None of these compounds combines the 3β,25 hemiketal functionality, C-28 methyl group, and the C-19 isopropenyl group identified in compound **1**.

The infrared (IR) spectrum provided additional support for this structure (Figure S13). Compound **1** exhibited a characteristic broad absorption band at 3405 cm^−1^ (Figure S13), attributable to O–H stretching vibrations of a hydroxyl group attached to a cyclohexyl ring, consistent with the IR spectrum of lantabetulic acid (3372 cm^−1^) [32]. In contrast to this compound, which showed a strong C=O absorption around 1700 cm^−1^, no such band was observed for compound **1**, supporting the absence of a carboxylic acid group as indicated by the NMR data. Several intense absorption bands in the 1215–1174 cm^−1^ region were assigned to C–O–C stretching vibrations, consistent with the presence of a hemiketal functionalty.

**Figure 2:**
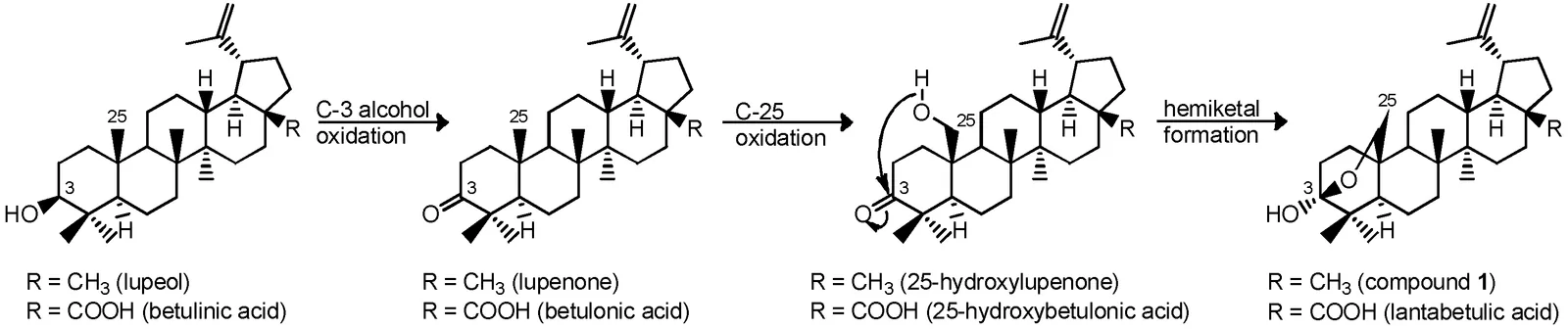
Hypothetical biosynthetic pathways proposed for lantabetulic acid and compound **1** in *Rhus* spp., based on structural analogy.

From a biosynthetic perspective, compound **1** may be structurally related to other lupane-type triterpenoids reported in *Rhus* spp., such as betulinic acid, betulonic acid, and lantabetulic acid, which have been isolated from various organs [22]. The formation of lantabetulic acid may involve sequential oxidation of betulinic acid at C-3 to yield betulonic acid, followed by hydroxylation at C-25 and subsequent cyclization to form the hemiketal structure [42]. By analogy, the biosynthesis of compound **1** could hypothetically proceed from lupeol, a triterpenoid previously reported in *Rhus* spp. [39], via lupenone, followed by further oxidative transformations and cyclization (Figure 2). These proposed pathways remain hypothetical and have not been experimentally verified.

**Figure 3:**
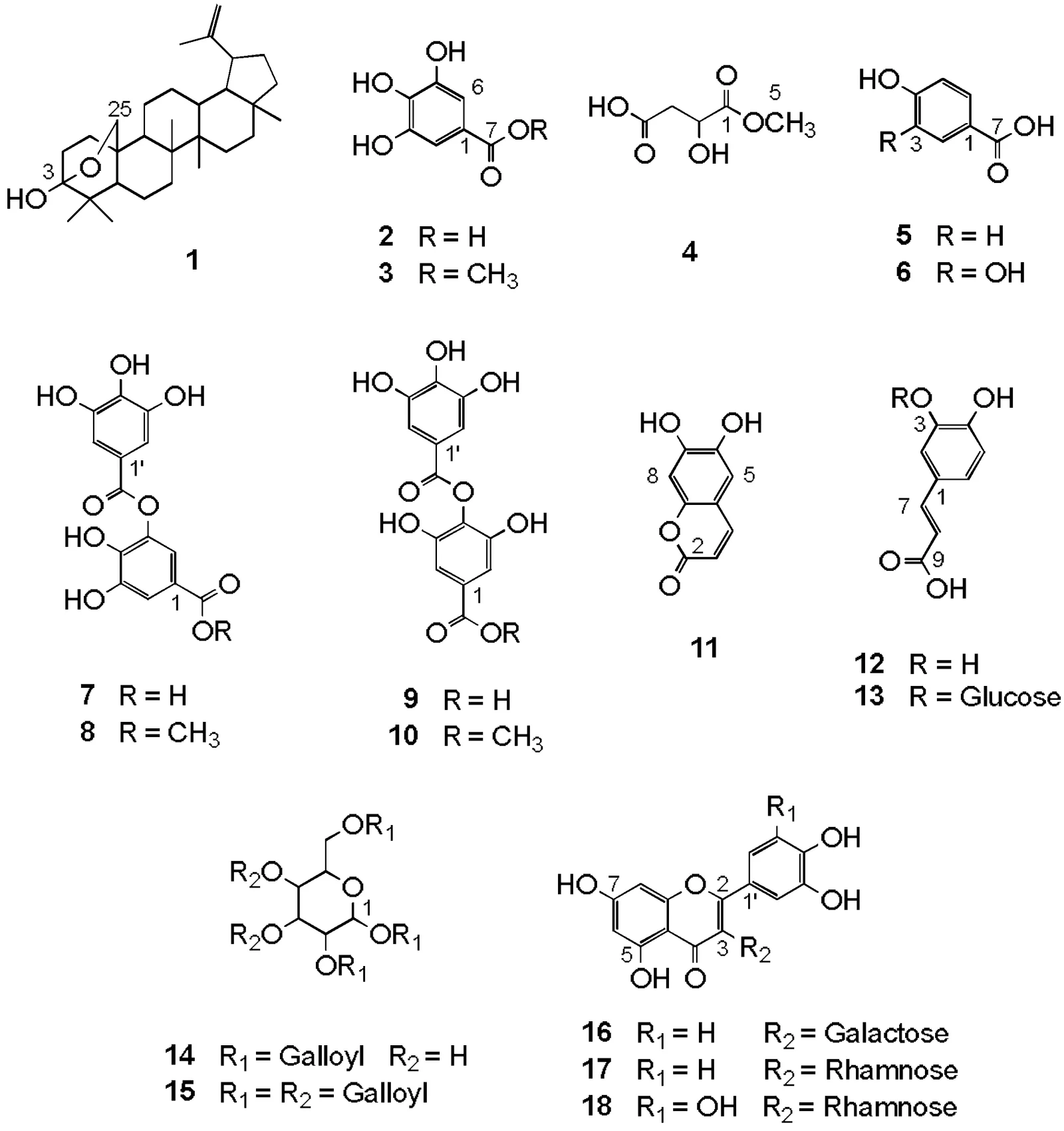
Structures of compounds (**1**–**18**) isolated from the drupes of *R. typhina.*

The other isolated compounds (**2**–**18**) were identified by comparing their spectroscopic data obtained in this study, including NMR and HRMS data, where applicable, with previously published data. These compounds included gallic acid (**2**), methyl gallate (**3**), 1-methyl malate (**4**), 4-hydroxybenzoic acid (**5**), protocatechuic acid (**6**), *m*-digallic acid (**7**), methyl *m*-digallate (**8**), *p*-digallic acid (**9**), methyl *p*-digallate (**10**), aesculetin (**11**), caffeic acid (**12**), 3-*O*-β-d-glucopyranosyl-*trans*-caffeic acid (**13**), 1,2,6-tri-*O*-galloyl-β-d-glucopyranose (**14**), 1,2,3,4,6-penta-*O*-galloyl-β-d-glucopyranose (**15**), hyperoside (**16**), quercitrin (**17**), and myricitrin (**18**) (Figure 3).

Previous studies have reported gallic acid (**2**), methyl gallate (**3**), caffeic acid (**12**) [21], and quercitrin (**17**) [43] in *R. typhina* fruits. Compound **13**, identified here as 3-*O*-β-d-glucopyranosyl-*trans*-caffeic acid may have been previously reported as caffeic acid 3-*O*-hexose [21,43], based on MS analysis (without definitive identification of the sugar moiety). Other compounds have been reported in different plant parts or species of the genus. These include protocatechuic acid (**6**) and 1,2,3,4,6-penta-*O*-galloyl-β-D-glucose (**15**) in *R. typhina* leaves [10,44], 4-hydroxybenzoic acid (**5**) in *R. chinensis* [22], hyperoside (**16**) in *R. coriaria* [45] and *R. chinensis* [46], and myricitrin (**18**) in *R. tripartita* [47]. To the best of our knowledge, 1-methyl malate (**4**) has not previously been reported in *Rhus*, although a galloylated derivative of dimethyl malate was identified in *R. copallinum* fruits [48]. The identification of *m*-digallic acid (**7**) is consistent with previous reports of digallic acid isomers in *R. coriaria* [49,50] and *R. chinensis* [23], although the ester linkage position were not specified. Methyl esters of *m*-digallic acid (**8**) and *p*-digallic acid (**10**) were previously identified in *R. copallinum* [48] but not in *R. typhina*. Aesculetin (**11**) is reported here for the first time in the genus, although its methylated derivative, scoparone, was previously found in *R. typhina* fruits [21]. Finally, to the best of our knowledge, 1,2,6-tri-*O*-galloyl-β-D-glucose (**14**) has not previously been reported as a constituent of *Rhus* species. However, it has been described as an intermediate in gallotannin biosynthesis using enzymes from *R. typhina* leaves [51]. Its positional isomer, 1,4,6-tri-*O*-galloyl-β-D-glucose, has also been reported in *R. typhina* leaves [10].

### 2.3. Antibacterial Activity of Compounds

Among the 18 compounds isolated from *R. typhina*, six were selected for biological evaluation based on the availability of sufficient amounts of material and the purity of the isolated compounds. Their antibacterial activity against *E. coli* and *S. aureus* and their cytotoxicity toward the healthy human fibroblast cell line WS-1 are summarized in Table 3.

None of the six isolated compounds tested showed measurable antibacterial activity against *E. coli* within the concentration ranges evaluated, whereas the methanolic extract and several fractions were active. This difference could reflect the contribution of untested constituents, interactions among constituents, or differences in compound solubility and availability under the assay conditions. These explanations remain hypotheses, as they were not directly investigated in this study. Further bioassay-guided fractionation and evaluation of additional constituents would help identify the compounds responsible for the observed activity. Compound-combination experiments would be required to assess potential synergistic effects, while solubility controls and testing under matched assay conditions would help clarify the contribution of compound availability. The lower susceptibility of Gram-negative bacteria such as *E. coli* to hydrophobic natural products may be related to the permeability barrier imposed by the outer membrane [52,53].

In contrast, two of the selected compounds exhibited antibacterial activity against *S. aureus.* Compound **1** was the most active compound, with an IC_50_ of 0.052 ± 0.004 µM. Hyperoside (**16**) also showed antibacterial activity, with an IC_50_ of 0.48 ± 0.09 µM. Thus, compound **1** was approximately nine times more active than hyperoside under the conditions tested. Their cytotoxicity (IC_50_) values against WS-1 fibroblasts were >100 µM, indicating that a 50% reduction in the resazurin-based viability signal was not achieved at the highest concentration tested. This assay provided a preliminary assessment of cytotoxicity within the tested concentration range. Further evaluation in additional cellular models would be required to characterize its safety profile. Previous studies have reported antibacterial effects of hyperoside (**16**) against several bacterial species, including *P. aeruginosa* and *Shewanella putrefaciens*, generally at substantially higher concentrations [54], as well as effects on *P. aeruginosa* biofilm formation and quorum sensing [55].

Although compounds **1** and **16** showed activity against *S. aureus*, their individual activities cannot fully account for the antibacterial activity observed in the crude extract. These findings suggest that different constituents may contribute to the antibacterial activity of the extract, depending on the bacterial species. Further evaluation using additional bacterial strains and complementary biological assays would help clarify the respective roles of individual constituents in the antibacterial activity of the extract.

Structure–activity relationship studies indicate that the antibacterial activity of pentacyclic triterpenoids is influenced by the nature and position of functional groups on the triterpene scaffold, with modifications at C-3 and C-28 playing a particularly important role [56]. The β-orientation of the C-3 hydroxyl group in oleanane derivatives has also been associated with antibacterial activity [57], while the presence of additional oxygenated functionalities has been associated with differences in antibacterial activity among triterpenoids [58]. Chemical derivatization of pentacyclic triterpenoids can substantially alter antibacterial activity [1]. In this context, the low IC_50_ of compound **1** suggests that its unusual structural features may contribute to its antibacterial activity. In particular, the intramolecular hemiketal bridge between C-3 and C-25 is uncommon among pentacyclic triterpenoids and has rarely been investigated in relation to antibacterial activity [59,60]. However, no direct structure–activity relationship can be established from the present data. Dedicated comparison with closely related analogues will be required to determine whether the hemiketal motif contributes to the antibacterial effect.

More broadly, triterpenoids remain relatively rare in pharmaceutical development, with only a few compounds reaching clinical approval to date, in part due to challenges in synthesis, purification, and optimization of their pharmacological profiles [61]. Nevertheless, notable exceptions exist. In particular, enfumafungin, a triterpenoid natural product isolated from a fungal source, led to the development of ibrexafungerp, the first approved triterpenoid antifungal agent [62,63]. Interestingly, enfumafungin contains an ether bridge in ring A, and despite the difference in functional groups (hemiketal vs. ether bridge), both molecules contain structural elements within the triterpenoid scaffold that could influence molecular conformation. This example highlights that structurally distinctive triterpenoids can give rise to clinically useful compounds. In this context, the potential contribution of the hemiketal bridge in compound **1** to its biological activity remains to be elucidated and would require dedicated structure–activity relationship studies.

Overall, this study demonstrated the antibacterial activity of *R. typhina* drupes. Compound **1**, a new lupane-type triterpenoid, exhibited sub-micromolar inhibitory activity against *S. aureus*. Hyperoside (**16**) also showed measurable antibacterial activity against *S. aureus*. These findings further support *R. typhina* as a source of structurally diverse bioactive metabolites and identify compound **1** as an interesting candidate for further antibacterial characterization using additional bacterial strains and complementary biological assays. Indeed, the antibacterial assessment was limited to two bacterial strains. Further studies involving a broader panel of bacterial strains, including both Gram-positive and Gram-negative bacteria, are needed to determine whether the observed antibacterial activity extends to other bacterial species.

## 3. Materials and Methods

### 3.1. General Experimental Procedures

MeOH, chloroform (CHCl_3_), acetone, and EtOAc, all ACS grade and used for extraction, were purchased from Fisher Chemical^TM^ (Pittsburg, PA, USA). Ultrapure water (resistivity, 18.2 MΩ·cm) was obtained from a Milli-Q system (MilliporeSigma, Burlington, MA, USA) equipped with a 0.22 µm final filter. MeOH (HPLC Optima grade), formic acid (HPLC Optima grade), CD_3_OD, and CDCl_3_ were purchased from Fisher Chemical^TM^.

A Universal Cutting Mill (pulverisette 19, No. 19.1850.00) equipped with a 10 L drip tray and a P-19 V2A sieve cassette (4 mm square perforation) was purchased from Fritsch (Idar-Oberstein, Germany). Ultrasonic extraction was performed in a Bransonic ultrasonic cleaner (model 8510R-DTH, Branson Ultrasonic Corporation, Danbury, CT, USA).

Flash chromatography was performed on a Pure C-815 Flash system (Büchi, New Castle, DE, USA) equipped with a fraction collector and a UV detector. A reversed-phase C18 column (SiliCycle^TM^ SiliaSep^TM^ Flash, 120 g, 17% carbon, 40–63 µm particle size, 60 Å pore size, 36 × 256 mm) was used for flash separations. Solid-phase extraction (SPE) cartridges (InnoSep™, C18/14% carbon, 1000 mg/6 mL) were purchased from Canadian Life Sciences (Peterborough, ON, Canada). Semi-preparative HPLC purifications were carried out on a Shimadzu system (Shimadzu Scientific Instruments, Columbia, MD, USA) comprising a degasser (DGU-20A5R), a 2 mL injector (SIL-10AP), a communication module (CBM-20A), a UV diode-array detector (SPD-M20A), a fluorescence detector (RF-20A), and a fraction collector (FRC-10A). A Kinetex C18 column (5 µm, 100 Å, 250 × 21.2 mm; Phenomenex, Torrance, CA, USA) was used for semi-preparative purifications.

Specific optical rotations ([α]^20^_d_) of purified compounds were measured at 589 nm (20 °C) with an Autopol^®^ IV automatic polarimeter (Rudolph Research Analytical, Hackettstown, NJ, USA) equipped with a 6 mL cell (type 14). NMR spectra (^1^H, ^13^C, DEPT-135, DQF-COSY, HSQC, HMBC, and NOESY) were recorded on a Bruker Ascend NEO 500 MHz spectrometer (125.8 MHz for ^13^C, 500.3 MHz for ^1^H) equipped with a Prodigy broadband cryoprobe (Bruker, Billerica, MA, USA). Chemical shifts are reported in ppm (δ) with the residual solvent peaks as references (δ_H_: 3.31; δ_C_: 49.00 for CD_3_OD and δ_H_: 7.26; δ_C_: 77.16 for CDCl_3_) [64]. The infrared spectrum was recorded on a Cary 630 FT-IR spectrometer (Agilent Technologies, Santa Clara, CA, USA) using a NaCl pellet. High-resolution mass spectrometry (HRMS) analysis was performed on an Agilent 6546 ESI-Q-TOF system coupled to an Agilent 1260 Infinity II LC system. Low-resolution mass spectrometry was performed on an Agilent 1260 Series HPLC system coupled to an MSD iQ spectrometer.

### 3.2. Plant Material

*R. typhina* drupes were collected in August 2019 in the Saguenay–Lac-Saint-Jean region (Quebec, Canada). The plant was identified by Luc Godin (Biologist, Champignon Boréal), and a voucher specimen (No. QFA0674405) was deposited at the Louis-Marie Herbarium, Université Laval (Québec, Canada). After collection, the drupes were initially air-dried on trays for three weeks. Then they underwent a second drying phase in a ventilated oven at 40 °C for 48 h, spread in a thin layer. Finally, the dried drupes were ground using a cutting mill.

### 3.3. Extraction and Purification

A total of 1.02 kg of dried and ground *R. typhina* drupes were extracted with MeOH at a 1:15 ratio (*w*/*v*) using an ultrasonic bath (250 W, 30 °C) for 30 min. This procedure was repeated three times. After each extraction, the solvent was recovered and filtered through a Büchner funnel with a Whatman grade 4 filter paper. The combined methanolic fractions were then evaporated to dryness, yielding 252 g of a sticky red crude residue (24.7%, *w*/*w*, based on the initial dry plant material).

For liquid–liquid partitioning, 225 g of the methanolic extract was processed in portions, each suspended in Milli-Q water at a ratio of 1:5 (*w*/*v*). Each portion was extracted five times with EtOAc/H_2_O (5:1, *v*/*v*). The combined organic and aqueous phases were separately evaporated to dryness, yielding an EtOAc fraction (117.9 g, 52.4% *w*/*w*) and an aqueous fraction (104.1 g, 46.2% *w*/*w*). The two fractions accounted for 98.7% recovery, with 1.3% lost during partitioning.

A portion of the EtOAc fraction was subjected to fractionation on Diaion HP-20 resin (1.0 kg) at a sample-to-resin ratio of 1:50, using three successive batches of approximately 20 g each (59.5 g in total). The column was eluted with a H_2_O/MeOH gradient: 1.5 L of 45% MeOH, 2.0 L of 55% MeOH, 2.0 L of 70% MeOH, and 2.0 L at 100% MeOH, followed by washing with EtOAc. This procedure yielded fractions A (eluted with 45% MeOH; 18.6 g, 31.3%), B (eluted with 55% MeOH; 13.2 g, 22.2%), C (eluted with 70% MeOH; 19.6 g, 32.9%), D (eluted with 100% MeOH followed by 100% EtOAc; 7.4 g, 12.4%). The four fractions accounted for 98.8% recovery, with 1.2% lost during fractionation.

Fraction A was further separated on Diaion HP-20 (ratio 1:25) using a H_2_O/MeOH gradient (55:45 to 0:100), yielding subfractions A.1–A.4. Subfraction A.2 (1.0 g) was separated by automated flash chromatography (H_2_O/MeOH 80:20 to 60:40, 20 mL/min, 78 min) producing six subfractions (A.2.1–A.2.6). Subfraction A.2.1 (t_R_ = 10.0 min, 150.0 mg) was separated on semi-preparative HPLC (H_2_O/MeOH 85:15 to 80:20 in 32 min, 20 mL/min) yielding compound **4** (t_R_ = 4.0 min, 12.0 mg). Subfraction A.2.2 (t_R_ = 16.0 min, 100.0 mg) was separated on semi-preparative HPLC (H_2_O/MeOH 85:15 to 70:30 in 35 min, 20 mL/min) yielding compound **6** (t_R_ = 10.2 min, 3.9 mg). Subfraction A.2.3 (t_R_ = 21.0 min, 120.0 mg) was separated on semi-preparative HPLC (H_2_O/MeOH 85:15 to 65:35 in 45 min, 20 mL/min) yielding compounds **3** (t_R_ = 16.5 min, 1.9 mg), **13** (t_R_ = 20.5 min, 19.1 mg), **14** (t_R_ = 34.5 min, 0.8 mg), together with co-eluting compounds **7** and **8** (t_R_ = 14.0 min, 5.5 mg). Subfraction A.3 (200.0 mg) was separated on semi-preparative HPLC (H_2_O/MeOH 75:25 to 65:35 in 45 min, 20 mL/min) yielding compounds **3** (t_R_ = 3.0 min, 11.9 mg), co-eluting compounds **5** and **11** (t_R_ = 9 min, 3.3 mg), **12** (t_R_ = 10.5 min, 8.2 mg), and **15** (t_R_ = 27.0 min, 35.6 mg). Subfraction A.4 (2.0 g) was separated by automated flash chromatography (H_2_O/MeOH 74:26 to 55:45 in 97 min, 20 mL/min) producing seven subfractions. Subfraction A.4.7 (t_R_ = 92 min, 220.0 mg) was purified on normal-phase silica gel with 55:15:1 CHCl_3_/MeOH/H_2_O followed by 26:14:3 CHCl_3_/MeOH/H_2_O gradients to isolate co-eluting compounds **9** and **10** (3.3 mg).

Fraction B was separated by exclusion chromatography on Sephadex LH-20 (ratio 1:10). A H_2_O/MeOH gradient (55:45 to 0:100) eluted phenolic compounds, followed by a 50:50 H_2_O/acetone mixture to elute more strongly retained phenolic constituents [65]. Subfractions B.1 (96.2 mg) and B.2 (53.6 mg) were separated individually on semi-preparative HPLC (H_2_O/MeOH 90:10 to 50:50 in 40 min, 20 mL/min). Subfraction B.1 yielded compound **13** (t_R_ = 17.2 min, 1.9 mg), and B.2 yielded compounds **3** (t_R_ = 12.5 min, 18.7 mg) and **12** (t_R_ = 17.3 min, 3.1 mg). Subfraction B.3 (46.2 mg) was separated on semi-preparative HPLC (H_2_O/MeOH 86:14 to 40:60 in 30 min, 20 mL/min) yielding compounds **2** (t_R_ = 5.0 min, 0.8 mg), **16** (t_R_ = 23.5 min, 36.5 mg), **17** (t_R_ = 26.0 min, 1.9 mg), as well as co-eluting compounds **9** and **10** (t_R_ = 26.5 min, 2.8 mg). Subfraction B.4 (106.6 mg) was separated on semi-preparative HPLC (H_2_O/MeOH 80:20 to 50:50 in 35 min, 20 mL/min) yielding compound **18** (t_R_ = 16.3 min, 7.9 mg).

Fraction D was separated on normal-phase silica gel (ratio 1:25) using a 50:1 CHCl_3_/MeOH eluent, yielding subfractions D.1–D.7. Several subfractions were obtained, including D.3 (1.78 g), which was further purified on silica gel using 100% CHCl_3_ to yield subfraction D.3.3 (328.5 mg). Compound **1** (46.3 mg) was finally isolated from this subfraction using a 5:1 hexane/EtOAc mixture on silica gel.

### 3.4. Structural Elucidation of Isolated Compounds

3α-hydroxy-3β,25-epoxylup-20(29)-ene (**1**): pale yellow crystalline powder; [α]^20^_d_ –36.5° (c = 0.7, CHCl_3_); HR-ESI-MS (positive ion mode), *m*/*z* 441.3710 [M+H]^+^ (calcd for C_30_H_49_O_2_^+^: 441.3727, ∆ = 3.87 ppm); IR *ν*_max_ (NaCl, cm^−1^): 3405 (br), 2930, 1457, 1380, 1215, 1174, 1065, 1015, 884, 758 (see Figure S13); ^1^H and ^13^C NMR data (CDCl_3_): see Table 2 and Table S1 and Figures S3–S12.

Gallic acid (**2**): white amorphous powder; MS (negative ion mode), *m*/*z* 169 [M−H]^−^; ^1^H NMR (CD_3_OD) δ: 7.05 (2H, s, H-2,6); ^13^C NMR (CD_3_OD) δ: 170.4 (C-7), 146.4 (C-3,5), 139.6 (C-4), 122.0 (C-1), 110.4 (C-2,6); NMR data are consistent with those reported by Sen et al. [66].

Methyl gallate (**3**): white amorphous powder; MS (negative mode), *m*/*z* 183 [M−H]^−^; ^1^H NMR (CD_3_OD) δ: 7.05 (2H, s, H-2,6), 3.81 (3H, s, H-8); ^13^C NMR (CD_3_OD) δ: 169.1 (C-7), 146.5 (C-3,5), 139.8 (C-4), 121.5 (C-1), 110.1 (C-2,6), 52.4 (C-8); NMR data are consistent with those reported by Fukuda et al. [67].

1-Methyl malate (**4**): light yellow powder; MS (negative ion mode), *m*/*z* 133 [M−H]^−^; ^1^H NMR (CD_3_OD) δ: 4.50 (1H, dd, *J* = 7.3, 4.7 Hz, H-2), 3.74 (3H, s, H-5), 2.78 (1H, dd, *J* = 16.1, 4.6 Hz, H-3a), 2.67 (1H, dd, *J* = 16.1, 7.3 Hz, H-3b). ^13^C NMR (CD_3_OD) δ: 175.2 (C-1), 173.9 (C-4), 68.7 (C-2), 52.7 (C-5), 40.0 (C-3); NMR data are consistent with those reported by He et al. [68].

4-Hydroxybenzoic acid (**5**): identified by ^1^H and ^13^C NMR analysis of a mixture of two compounds. ^1^H NMR (CD_3_OD) δ: 7.87 (2H, d, *J* = 8.8 Hz, H-2,6), 6.81 (2H, d, *J* = 8.8 Hz, H-3,5). ^13^C NMR (CD_3_OD) δ: 170.2 (C-7), 163.4 (C-4), 133.0 (C-2,6), 122.9 (C-1), 116.0 (C-3,5); NMR data are consistent with those reported by Mita et al. [69].

Protocatechuic acid (**6**): white amorphous powder; MS (negative ion mode), *m*/*z* 153 [M−H]^−^; ^1^H NMR (CD_3_OD) δ: 7.44–7.41 (2H, m, H-2,6), 6.80 (1H, d, *J* = 8.1 Hz, H-5); ^13^C NMR (CD_3_OD) δ: 170.3 (C-7), 151.6 (C-4), 146.1 (C-3), 123.9 (C-6), 123.2 (C-1), 117.8 (C-2), 115.8 (C-5); NMR data are consistent with those reported by Ban et al. [70].

*m*-Digallic acid (**7**) and *p*-digallic acid (**9**) were isolated as a mixture. Compound **7**: ^1^H NMR (CD_3_OD) δ: 7.40 (1H, d, *J* = 1.9 Hz, H-6), 7.26 (1H, d, *J* = 2.0 Hz, H-2), 7.22 (2H, s, H-2′,6′); ^13^C NMR δ (CD_3_OD): 169.6 (C-7), 166.7 (C-7′), 147.5 (C-5), 146.7 (C-4′), 144.2 (C-4), 140.5 (C-3′,5′), 140.1 (C-3), 122.2 (C-1), 120.6 (C-1′), 117.5 (C-2), 115.1 (C-6), 110.8 (C-2′,6′). Compound **9**: ^1^H NMR (CD_3_OD) δ: 7.24 (2H, s, H-2′,6′), 7.12 (2H, s, H-2,6); ^13^C NMR (CD_3_OD) δ: 169.6 (C-7), 166.3 (C-7′), 151.7 (C-3,5), 146.6 (C-4′), 140.4 (C-3′,5′), 132.7 (C-4), 129.7 (C-1), 120.7 (C-1′), 110.0 (C-2′,6′), 110.1 (C-2,6); NMR data are in agreement with those reported by Verzele et al. [71].

Methyl *m*-digallate (**8**) and methyl *p*-digallate (**10**) were isolated as a mixture. Compound **8**: ^1^H NMR (CD_3_OD) δ: 7.38 (1H, d, *J* = 1.9 Hz, H-6), 7.26 (1H, d, *J* = 2.0 Hz, H-2), 7.21 (2H, s, H-2′,6′), 3.85 (3H, s, H-8); ^13^C NMR (CD_3_OD) δ: 168.2 (C-7), 166.6 (C-7′), 147.7 (C-5), 146.7 (C-4′), 144.5 (C-4), 140.2 (C-3′, 5′,3), 121.4 (C-1), 120.4 (C-1′), 117.3 (C-2), 114.7 (C-6), 110.7 (C-2′,6′). Compound **10**: ^1^H NMR (CD_3_OD) δ: 7.22 (2H, s, H-2′,6′), 7.10 (2H, s, H-2,6), 3.87 (3H, s, H-8); ^13^C NMR (CD_3_OD) δ: 168.2 (C-7), 166.2 (C-7′), 151.8 (C-3,5), 146.6 (C-4′), 140.5 (C-3′,5′), 132.8 (C-4), 128.9 (C-1), 120.4 (C-1′), 110.9 (C-2,6). NMR data are in agreement with those reported by Fogagnolo et al. [72].

Aesculetin (**11**): identified by ^1^H and ^13^C NMR analysis of a mixture of two compounds. ^1^H NMR (CD_3_OD) δ: 7.78 (1H, d, *J* = 9.4 Hz, H-4), 6.94 (1H, s, H-5), 6.75 (1H, s, H-8), 6.18 (1H, d, *J* = 9.4 Hz, H-3); ^13^C NMR (CD_3_OD) δ: 164.3 (C-2), 152.0 (C-7), 150.5 (C-9), 146.1 (C-4), 144.6 (C-6), 113.1 (C-5), 112.8 (C-10), 112.5 (C-3), 103.7 (C-8); NMR data are consistent with those reported by Liu et al. [73].

Caffeic acid (**12**): white amorphous powder; MS (negative ion mode), *m*/*z* 179 [M−H]^−^; ^1^H NMR (CD_3_OD) δ: 7.53 (1H, d, *J* = 15.9 Hz, H-7), 7.04 (1H, d, *J* = 2.1 Hz, H-2), 6.93 (1H, dd, *J* = 8.2, 2.1 Hz, H-6), 6.78 (1H, d, *J* = 8.1 Hz, H-5), 6.22 (1H, d, *J* = 15.8 Hz, H-8); ^13^C NMR (CD_3_OD) δ: 171.1 (C-9), 149.5 (C-4), 147.0 (C-7), 146.8 (C-3), 127.9 (C-1), 122.9 (C-6), 116.5 (C-5), 115.6 (C-8), 115.1 (C-2); NMR data are consistent with those reported by Lavoie et al. [29].

3-*O*-*β*-d-glucopyranosyl-*trans*-caffeic acid (**13**): white amorphous powder; [α]^20^_d_ –43.8° (c = 0.3, MeOH); MS (negative ion mode), *m*/*z* 341 [M−H]^−^; ^1^H NMR (CD_3_OD) δ: 7.58 (1H, d, *J* = 15.9 Hz, H-7), 7.51 (1H, d, *J* = 2.1 Hz, H-2), 7.17 (1H, dd, *J* = 8.4, 2.0 Hz, H-6), 6.87 (1H, d, *J* = 8.3 Hz, H-5), 6.32 (1H, d, *J* = 15.9 Hz, H-8), 4.83 (1H, d, *J* = 7.3 Hz, H-1′), 3.94 (1H, dd, *J* = 12.1, 2.3 Hz, H-6′a), 3.72 (1H, dd, *J* = 12.1, 6.0 Hz, H-6′b), 3.55–3.46 (3H, m, H-2′,3′,5′), 3.40 (1H, m, H-4′); ^13^C NMR (CD_3_OD) δ: 171.0 (C-9), 151.0 (C-4), 147.1 (C-3), 146.5 (C-7), 128.1 (C-1), 126.0 (C-6), 118.1 (C-2), 117.5 (C-5), 116.6 (C-8), 104.3 (C-1′), 78.5 (C-5′), 77.6 (C-3′), 74.9 (C-2′), 71.5 (C-4′), 62.5 (C-6′). NMR data are consistent with those reported by Li et al. [22].

1,2,6-tri-*O*-galloyl-*β*-d-glucopyranose (**14**): white amorphous powder; [α]^20^_d_ –133.3° (c = 0.1, MeOH); MS (negative ion mode), *m*/*z* 635 [M−H]^−^; ^1^H NMR (CD_3_OD) δ: 7.11, 7.06, 7.02 (each 2H, s, H-2,6), 5.93 (1H, d, *J* = 8.4 Hz, H-1), 5.22 (1H, t, *J* = 8.9 Hz, H-2), 4.58 (1H, dd, *J* = 12.1, 2.0 Hz, H-6a), 4.48 (1H, dd, *J* = 12.1, 4.7 Hz, H-6b), 3.87–3.80 (2H, m, H-3,5), 3.68 (1H, t, *J* = 9.3 Hz, H-4); ^13^C NMR (CD_3_OD) δ: 168.3, 167.7, 166.6 (3 x galloyl C-7), 146.6, 146.5, 146.5 (3 x galloyl C-3,5), 140.6, 140.1, 140.0 (3 x galloyl C-4), 121.3, 121.1, 120.1 (3 x galloyl C-1), 110.6, 110.4, 110.3 (3 x galloyl C-2,6), 94.2 (C-1), 76.7 (C-5), 76.0 (C-3), 74.3 (C-2), 71.4 (C-4), 64.3 (C-6). NMR data are consistent with those reported by Lavoie et al. [29].

1,2,3,4,6-penta-*O*-galloyl-*β*-d-glucopyranose (**15**): light brown amorphous powder; [α]^20^_d_ +10.9° (c = 0.4, MeOH); MS (negative ion mode), *m*/*z* 939 [M−H]^−^; ^1^H NMR (CD_3_OD) δ: 7.12, 7.05, 6.98, 6.95, 6.90 (each 2H, s, galloyl H-2,6), 6.24 (1H, d, *J* = 8.3 Hz, glc. H-1), 5.91 (1H, t, *J* = 9.7 Hz, glc. H-3), 5.62 (1H, t, *J* = 9.6 Hz, glc. H-4), 5.59 (1H, dd, *J* = 9.9, 8.3 Hz, glc. H-2), 4.51 (1H, br d, *J* = 10.9 Hz, glc. H-6a), 4.44–4.36 (2H, m, glc. H-6b,5); ^13^C NMR (CD_3_OD) δ: 168.0, 167.3, 167.0, 167.0, 166.3 (5 x galloyl C-7), 146.6, 146.5, 146.5, 146.4, 146.3 (5 x galloyl C-3,5), 140.8, 140.4, 140.3, 140.2, 140.0 (5 x galloyl C-4), 121.1, 120.4, 120.3, 120.3, 119.8 (5 x galloyl C-1), 110.7, 110.5, 110.5, 110.4, 110.4 (5 x galloyl C-2,6), 93.9 (C-1), 74.5 (C-5), 74.2 (C-3), 72.2 (C-2), 69.8 (C-4), 63.2 (C-6); NMR data are consistent with those reported by Cho et al. [74].

Hyperoside (**16**): white amorphous powder; [α]^20^_d_ –2.1° (c = 0.4, MeOH); MS (negative ion mode), *m*/*z* 463 [M−H]^−^; ^1^H NMR (CD_3_OD) δ: 7.85 (1H, d, *J* = 2.2 Hz, H-2′), 7.59 (1H, dd, *J* = 8.5, 2.3 Hz, H-6′), 6.86 (1H, d, *J* = 8.5 Hz, H-5′), 6.41 (1H, d, *J* = 2.1 Hz, H-8), 6.20 (1H, d, *J* = 2.2 Hz, H-6), 5.18 (1H, d, *J* = 7.8 Hz, H-1″), 3.85 (1H, d, *J* = 3.4 Hz, H-4″), 3.82 (1H, dd, *J* = 9.8, 7.8 Hz, H-2″), 3.65 (1H, dd, *J* = 11.1, 6.0 Hz, H-6″a), 3.57 (2H, m, H-3″,6″b), 3.48 (1H, t, *J* = 6.1 Hz, H-5″); ^13^C NMR (CD_3_OD) δ: 179.6 (C-4), 166.1 (C-7), 163.1 (C-5), 158.8 (C-2), 158.5 (C-9), 150.0 (C-4′), 145.9 (C-3′), 135.8 (C-3), 122.9 (C-6′), 122.9 (C-1′), 117.8 (C-2′), 116.1 (C-5′), 105.6 (C-10), 105.3 (C-1″), 99.9 (C-6), 94.7 (C-8), 77.2 (C-5″), 75.1 (C-3″), 73.2 (C-2″), 70.0 (C-4″), 61.9 (C-6″); NMR data are consistent with those reported by Atay et al. [75].

Quercitrin (**17**): white amorphous powder; [α]^20^_d_ –117.7° (c = 0.1, MeOH); MS (negative ion mode), *m*/*z* 447 [M−H]^−^; ^1^H NMR (CD_3_OD) δ: 7.34 (1H, d, *J* = 2.1 Hz, H-2′), 7.31 (1H, dd, *J* = 8.3, 2.1 Hz, H-6′), 6.91 (1H, d, *J* = 8.3 Hz, H-5′), 6.38 (1H, d, *J* = 2.1 Hz, H-8), 6.20 (1H, d, *J* = 2.2 Hz, H-6), 5.35 (1H, d, *J* = 1.7 Hz, H-1″), 4.22 (1H, dd, *J* = 3.4, 1.7 Hz, H-2″), 3.75 (1H, dd, *J* = 9.4, 3.4 Hz, H-3″), 3.42 (1H, m, H-5″), 3.33 (1H, t, *J* = 9.4 Hz, H-4″), 0.94 (3H, d, *J* = 6.1 Hz, H-6″); ^13^C NMR (CD_3_OD) δ: 179.7 (C-4), 166.0 (C-7), 163.3 (C-5), 159.3 (C-2), 158.6 (C-9), 149.9 (C-4′), 146.5 (C-3′), 136.3 (C-3), 123.0 (C-1′), 122.9 (C-6′), 116.9 (C-2′), 116.4 (C-5′), 105.9 (C-10), 103.6 (C-1″), 99.8 (C-6), 94.7 (C-8), 73.2 (C-4″), 72.1 (C-5″), 72.1 (C-3″), 71.9 (C-2″), 17.7 (C-6″); NMR data are consistent with those reported by Yang et al. [76].

Myricitrin (**18**): white amorphous powder; [α]^20^_d_ –66.5° (c = 0.1, MeOH); MS (negative ion mode), *m*/*z* 463 [M−H]^−^; ^1^H NMR (CD_3_OD) δ: 6.95 (2H, s, H-2′,6′), 6.36 (1H, d, *J* = 2.1 Hz, H-8), 6.20 (1H, d, *J* = 2.0 Hz, H-6), 5.32 (1H, d, *J* = 1.7 Hz, H-1″), 4.22 (1H, dd, *J* = 3.4, 1.7 Hz, H-2″), 3.79 (1H, dd, *J* = 9.5, 3.4 Hz, H-3″), 3.52 (1H, m, H-5″), 3.34 (1H, t, *J* = 9.6 Hz, H-4″), 0.96 (3H, d, *J* = 6.2 Hz, H-6″); ^13^C NMR (CD_3_OD) δ: 179.7 (C-4), 165.9 (C-7), 163.3 (C-5), 159.5 (C-2), 158.5 (C-9), 146.9 (C-3′,5′), 137.9 (C-4′), 136.4 (C-3), 121.9 (C-1′), 109.6 (C-2′,6′), 105.9 (C-10), 103.7 (C-1″), 99.8 (C-6), 94.7 (C-8), 73.4 (C-4″), 72.2 (C-3″), 72.1 (C-5″), 71.9 (C-2″), 17.7 (C-6″); NMR data are consistent with those reported by Zhang et al. [77].

### 3.5. Antibacterial Activity

Antibacterial activity was evaluated against *Escherichia coli* (ATCC 25922) and *Staphylococcus aureus* (ATCC 25923) (ATCC, Manassas, VA, USA), with gentamicin (Sigma-Aldrich, St. Louis, MO, USA) used as a positive control. Two assays were used: a broth microdilution assay for the crude extract and fractions, and an agar-based assay for the isolated compounds. The broth microdilution assay was performed using a modified method [78,79]. Exponentially growing bacteria were distributed into 96-well microplates (Costar, Corning Inc., Corning, NY, USA) at densities of 5 × 10^3^ cells/well for *E. coli* and 3.5 × 10^4^ cells/well for *S. aureus*. Samples were dissolved in DMSO and diluted in nutrient broth (Difco™, BD, Sparks, MD, USA) to obtain the tested concentrations in a final volume of 200 µL per well. The final DMSO concentration was 0.25% (*v*/*v*). Plates were incubated at 37 °C for 24 h, and absorbance was measured at 600 nm using a Varioskan Ascent plate reader (Thermo Electron Corporation, Waltham, MA, USA). Solvent controls containing bacteria and 0.25% DMSO without test samples were included. The crude extract and fractions were screened in a single experiment with three technical replicate wells per concentration. These measurements were used to monitor antibacterial activity and guide the selection of fractions for further purification, rather than to assess reproducibility across independent experiments. The isolated compounds were evaluated using an agar-based assay previously developed and published by our group [80]. This method accommodates both hydrophobic and hydrophilic compounds. Briefly, 100 µL of nutrient agar was dispensed into each well of a 96-well microplate and allowed to solidify at room temperature for 1 h. Methanolic solutions containing increasing amounts of the test compounds or gentamicin were deposited onto the agar surface. Plates were maintained at room temperature for at least 2 h, until the methanol had completely evaporated. Bacterial suspensions were then added at 5 × 10^3^ cells in 20 µL. Plates were incubated at 37 °C for 5 h, after which 100 µL of resazurin sodium salt solution (50 µg/mL; Sigma-Aldrich, R-2127) was added to each well. Following an additional incubation of 2 h for *S. aureus* or 3 h for *E. coli*, fluorescence was measured using a Fluoroskan Ascent FL™ plate reader (Thermo Labsystems, Vantaa, Finland), with excitation and emission wavelengths of 530 and 590 nm, respectively. The active compounds were tested in at least two independent experiments, each with three technical replicate wells per concentration. Untreated bacterial cultures served as growth controls. For the agar-based assay, methanol controls were prepared by depositing and evaporating the same volume of methanol used for the test compounds before bacterial inoculation. These controls were tested in parallel to assess any effect of the solvent-deposition procedure on bacterial growth. Appropriate uninoculated blanks were included in both assays, and their signals were subtracted before normalization to the corresponding solvent control.

IC_50_ values were defined as the concentrations producing a 50% reduction in the blank-corrected assay signal relative to the corresponding solvent control. Values were determined by fitting concentration–response curves using a nonlinear sigmoidal regression model (four-parameter logistic model). For both assays, *n* = 3 denotes three technical replicate wells per concentration within a single experiment, not three independent experiments. No statistical comparisons were performed between samples.

### 3.6. In Vitro Cytotoxic Activity

Cytotoxicity was assessed in WS-1 (ATCC CRL-1502), a healthy human skin fibroblast cell line. Daunorubicin (Sigma-Aldrich) was used as a positive control. WS-1 cell line was cultured in minimum essential medium with the addition of Earle’s balanced salts, L-glutamine (Mediatech, Cellgro, Manassas, VA, USA), 10% fetal bovine serum (HyClone, Logan, UT, USA), vitamins (1x), penicillin (100 IU/mL), and streptomycin (100 µg/mL). The cells were incubated at 37 °C in a humidified atmosphere containing 5% CO_2_. The WS-1 cell line was transferred to 96-well microplates (Costar, Corning Inc) at a density of 5 × 10^3^ cells per well (100 µL) with culture medium and allowed to adhere for 16 h before treatment. Each sample was then diluted in DMSO to the desired concentrations and added to the wells (100 µL per well). The final concentration of DMSO in the culture medium was maintained at 0.25%. After a 48-h incubation period, resazurin (25 µg/mL in HBSS) was added to assess cytotoxicity. The fluorescence was measured at excitation at 530 nm and emission at 590 nm using a Fluoroskan Ascent FL™ automated 96-well microplate reader (Thermo Labsystems) as described by O’Brien et al. [81]. Fluorescence levels corresponded to cellular metabolic activity in each well. Cytotoxicity results were reported as mean ± standard deviation and expressed as the 50% inhibitory concentration (IC_50_) for cell growth and compared with the control (daunorubicin).

## 4. Conclusions

Phytochemical investigation of the drupes of *R. typhina* led to the isolation of eighteen compounds, including the previously undescribed lupane-type triterpenoid (**1**). Aesculetin (**11**) is reported for the first time in the genus *Rhus*, and several phenolic constituents were newly identified in *R. typhina*, further expanding its phytochemical profile. Compound **1** exhibited potent sub-micromolar inhibitory activity against *S. aureus* and a cytotoxicity IC_50_ >100 µM toward human WS-1 fibroblasts under the conditions tested. Hyperoside (**16**) also showed measurable antibacterial activity against *S. aureus*. Notably, no individual compound accounting for the antibacterial activity observed against *E. coli* was identified in the present study, indicating that additional fractionation and biological investigations will be required to determine the constituents responsible for this activity. Collectively, these findings highlight *R. typhina* as a source of structurally diverse bioactive metabolites and identify compound **1** as an interesting lupane-type compound for further antibacterial characterization. Further studies should investigate its mechanism of action and antibacterial spectrum using a broader panel of *S. aureus* strains, including antibiotic-resistant isolates.

## Figures and Tables

**Table 1:** Preliminary screening of the antibacterial activity of the methanolic extract of *R. typhina* drupes and its derived subfractions to guide subsequent fractionation.

Fraction	Antibacterial Activity IC_50_ (µg/mL) ^a^	
	*E. coli*	*S. aureus*
Methanolic extract	12 ± 1	33 ± 8
Aqueous fraction	127 ± 8	>200
Ethyl acetate fraction	7.7 ± 0.9	31 ± 8
Fraction A	22 ± 1	29 ± 5
Fraction B	5.6 ± 0.6	33 ± 6
Fraction C	7 ± 1	22 ± 9
Fraction D solubilized in H_2_O Fraction D solubilized in DMSO ^c^	>200 26 ± 6	>200 25 ± 2
Gentamicin ^b^	0.0272 ± 0.0003 (0.057 ± 0.001 µM)	0.029 ± 0.005 (0.061 ± 0.011 µM)

^a^ IC_50_ values are mean ± SD (*n* = 3); ^b^ Gentamicin was used as the positive control for antibacterial assays. ^c^ DMSO was used at a final concentration of <0.5% (*v*/*v*) in the culture medium. Note: Values are based on three technical replicate wells per concentration in a single experiment.

**Table 2:** ^1^H (500 MHz) and ^13^C (126 MHz) NMR data of compound **1** ^a^.

Position	δ_C_, Type	δ_H_ (mult., *J* in Hz)
1	35.3, CH_2_	2.18 (m), 1.10 (m)
2	29.7, CH_2_	2.13 (m), 1.67 (m)
3	98.9, C	–
4	40.5, C	–
5	49.9, CH	1.16 (m)
6	19.9, CH_2_	1.57 (m), 1.45 (m)
7	32.2, CH_2_	1.43 (m), 1.30 (m)
8	39.9, C	–
9	45.2, CH	1.42 (dd, 12.7, 2.7)
10	35.7, C	–
11	22.4, CH_2_	1.52 (m), 1.01 (m)
12	25.6, CH_2_	1.65 (m), 1.02 (m)
13	38.4, CH	1.66 (m)
14	42.7, C	–
15	27.6, CH_2_	1.61 (m), 1.07 (m)
16	35.6, CH_2_	1.47 (m), 1.36 (m)
17	43.2, C	–
18	48.2, CH	1.40 (m)
19	48.0, CH	2.36 (m)
20	150.9, C	–
21	30.0, CH_2_	1.92 (m), 1.31 (m)
22	40.1, CH_2_	1.38 (m), 1.19 (m)
23	27.0, CH_3_	1.01 (s)
24	18.5, CH_3_	0.96 (s)
25	68.2, CH_2_	4.24 (dd, 8.8, 2.9), 3.72 (dd, 8.8, 1.9)
26	16.4, CH_3_	0.94 (s)
27	14.4, CH_3_	0.92 (s)
28	18.2, CH_3_	0.77 (s)
29	109.5, CH_2_	4.68 (d, 2.4), 4.56 (dq, 2.7, 1.4)
30	19.5, CH_3_	1.67 (s)

^a^ Recorded in CDCl_3_. Chemical shifts (δ) are reported in ppm. Proton multiplicities and coupling constants (*J* in Hz) were assigned from the ^1^H NMR data.

**Table 3:** Antibacterial activity against *E. coli* and *S. aureus* and cytotoxicity toward WS-1 cells of *R. typhina* compounds selected for testing based on material availability and purity.

Compound	Antibacterial Activity IC_50_ (µM) ^a^		Cytotoxicity IC_50_ (µM) ^b^
	*E. coli*	*S. aureus*	WS-1
**1**	>5	0.052 ± 0.004	>100
**3**	>100	>100	>100
**6**	>100	>100	>100
**12**	>100	>100	>100
**15**	>100	>100	25 ± 2
**16**	>100	0.48 ± 0.09	>100
Gentamicin ^c^	0.034 ± 0.003	0.032 ± 0.007	(–)
Daunorubicin ^d^	(–)	(–)	<0.39

^a^ Antibacterial IC_50_ values are expressed as mean ± SD (*n* = 3); ^b^ Cytotoxicity IC_50_ values are expressed as mean ± SD (*n* = 3); ^c^ Gentamicin was used as the positive control for the antibacterial assays. ^d^ Daunorubicin was used as the positive control for cytotoxicity assay. Notes: Values are based on three technical replicate wells per concentration in a single experiment. Results for compounds **1** and **16** are representative of at least two independent experiments.

## Data Availability

Dataset available on request from the authors.

## Supplementary Materials

The following supporting information can be downloaded at: https://www.mdpi.com/article/10.3390/molecules31214012/s1, Figure S1: Normal-phase silica gel 60 F_254_ TLC profiles of the EtOAc fraction and its subfractions (A–D); Figure S2: HR-ESI-MS, positive ion mode mass spectrum of compound **1**; Table S1: ^1^H (500 MHz), ^13^C (126 MHz), HSQC, HMBC, COSY and NOESY NMR spectroscopic data for compound **1** (δ in ppm; recorded in CDCl_3_); Figure S3: ^1^H NMR spectrum (CDCl_3_, 500 MHz) of compound **1**; Figure S4: Enlarged view of ^1^H NMR spectrum (CDCl_3_, 500 MHz) of compound **1**; Figure S5: ^13^C NMR spectrum (CDCl_3_, 126 MHz) of compound **1**; Figure S6: ^13^C DEPT 135 NMR spectrum (CDCl_3_, 126 MHz) of compound **1**; Figure S7: ^1^H−^13^C HSQC NMR spectrum (CDCl_3_, 500 MHz) of compound **1**; Figure S8: Band-selective ^1^H–^13^C HSQC NMR spectrum (CDCl_3_, 500 MHz) of compound **1**; Figure S9: ^1^H–^1^H DQF COSY NMR spectrum (CDCl_3_, 500 MHz) of compound **1**; Figure S10: ^1^H–^13^C HMBC NMR spectrum (CDCl_3_, 500 MHz) of compound **1**; Figure S11: Band-selective ^1^H–^13^C HMBC NMR spectrum (CDCl_3_, 500 MHz) of compound **1**; Figure S12: ^1^H–^1^H NOESY NMR spectrum (CDCl_3_, 500 MHz) of compound **1**; Figure S13: IR spectrum of compound **1**.

## Abbreviations

The following abbreviations are used in this manuscript:

COSY	Correlation Spectroscopy
DEPT-135	Distortionless Enhancement by Polarization Transfer
DMSO	Dimethyl sulfoxide
DQF-COSY	Double Quantum Filtered Correlation Spectroscopy
EtOAc	Ethyl acetate
HMBC	Heteronuclear Multiple Bond Correlation
HPLC	High-Performance Liquid Chromatography
HRMS	High-Resolution Mass Spectrometry
HSQC	Heteronuclear Single Quantum Coherence
IC	Inhibitory concentration
IR	Infrared
MeOH	Methanol
MS	Mass Spectrometry
NMR	Nuclear Magnetic Resonance
NOESY	Nuclear Overhauser Effect Spectroscopy
TLC	Thin Layer Chromatography
