# Supplementary material for: Phytochemical Investigation of Rhus typhina Drupes and Identification of a New Antibacterial Lupane-Type Triterpenoid
Source: Molecules. 2026 Nov 6;31(21):4012. doi: 10.3390/molecules31214012 (PMC13764784; doi:10.3390/molecules31214012)
Supplement: Supplementary file 1 [file molecules-31-04012-s001.zip › molecules-4596248-supplementary.pdf]

Supplementary Materials

Phytochemical Investigation of *Rhus typhina* Drupes and Identification of a New Antibacterial Lupane-Type Triterpenoid

Marie Frissard [1†], Marianne Piochon [1†], Jean Legault [1], Vakhtang Mshvildadze [1], Nassim Djabou [1], Jérôme Alsarraf [1], Antoine Desportes [1], Josianne Savard [1] and André Pichette [1,*]

[1] Laboratoire d'Analyse et de Séparation des Essences Végétales, Centre de Recherche sur la Boréalie, Département des Sciences Fondamentales, Université du Québec à Chicoutimi, Saguenay, G7H 2B1, QC, Canada.

[†] These authors contributed equally to this work, * Correspondence: andre_pichette@uqac.ca

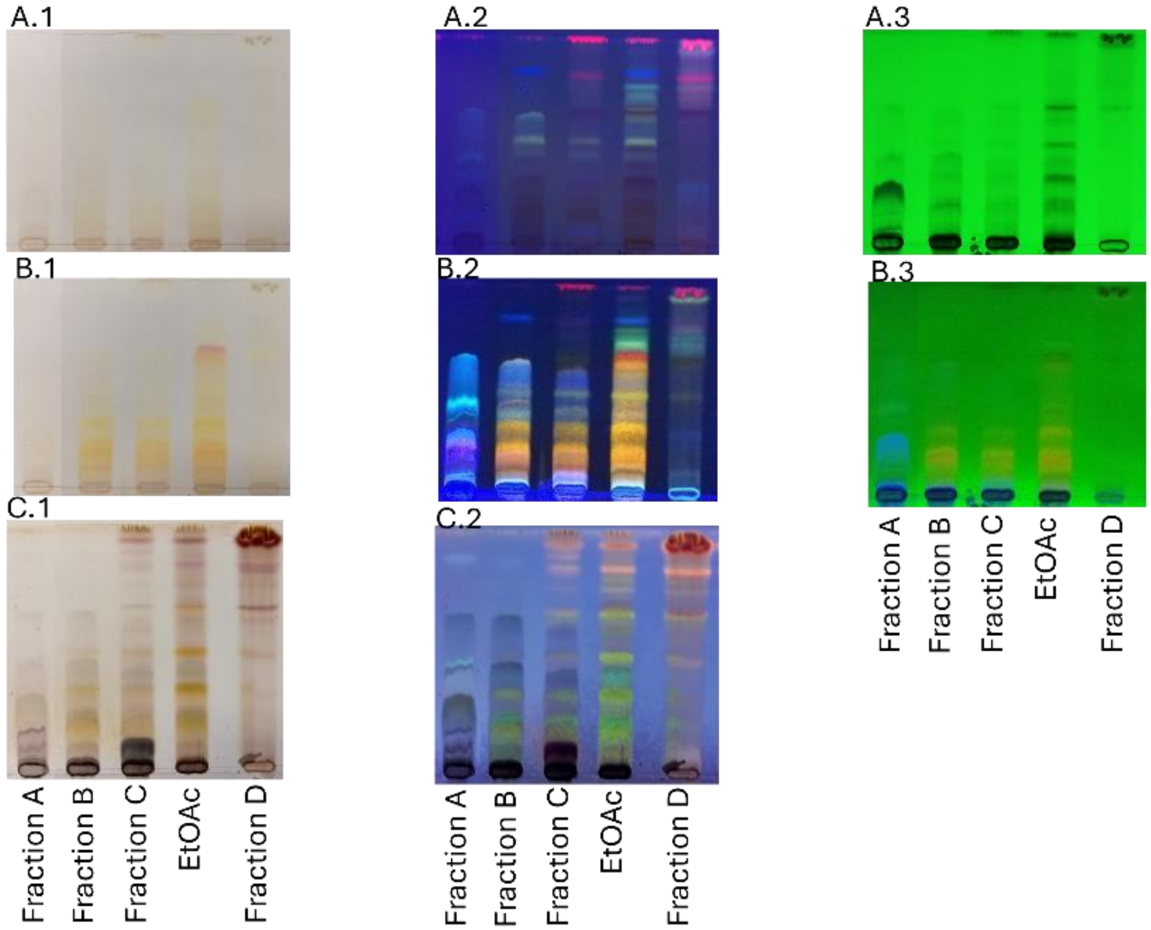

Figure S1. Normal-phase silica gel 60 F_{254} TLC profiles of the EtOAc fraction and its subfractions (A–D). (A) Untreated plate: (A.1) visible light, (A.2) UV light at 365 nm, and (A.3) UV light at 254 nm. (B) Plate after NP/PEG derivatization: (B.1) visible light, (B.2) UV light at 365 nm, and (B.3) UV light at 254 nm. (C) Plate after H_2SO_4 derivatization: (C.1) visible light and (C.2) UV light at 365 nm. Eluent: $CHCl_3$/MeOH/H_2O (55:15:1, v/v/v).

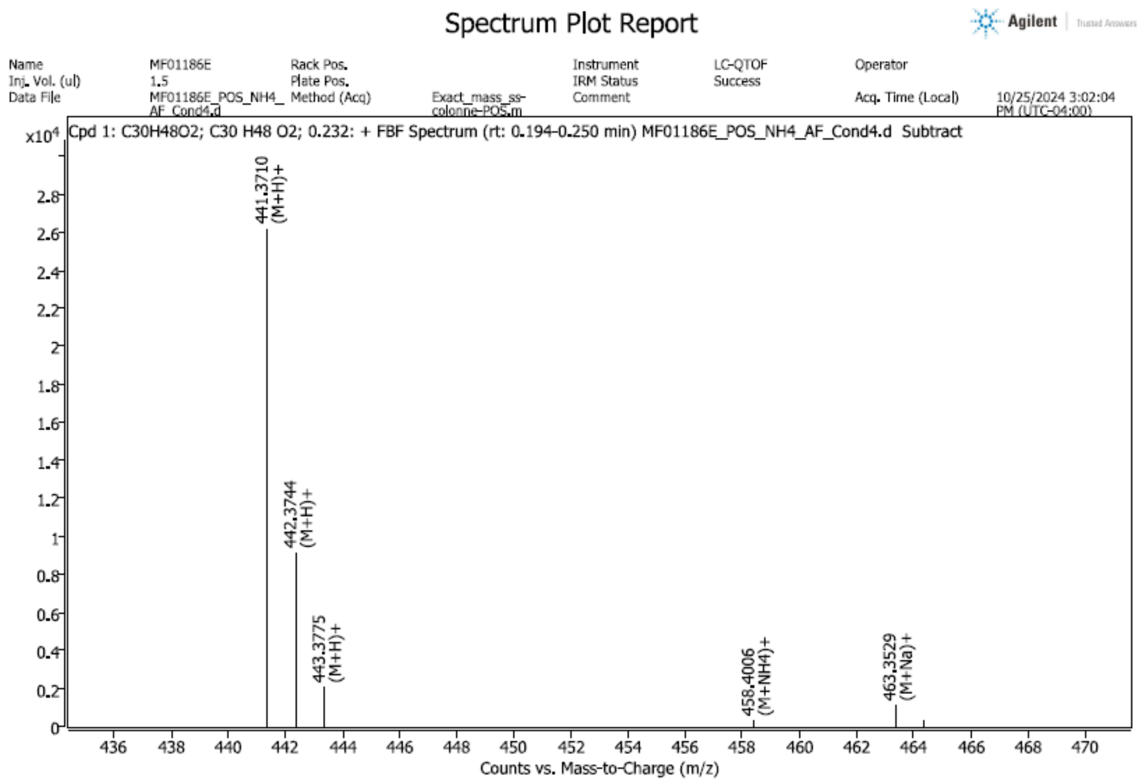

Figure S2. HR-ESI-MS, positive ion mode mass spectrum of compound **1.**

Table S1. ^{1}H (500 MHz), ^{13}C (126 MHz), HSQC, HMBC, COSY and NOESY NMR spectroscopic data for compound **1** (*δ* in ppm; recorded in $CDCl_3$).

Pos.	^{13}C $δ_C$, type	^{1}H $δ_H$ (mult., *J* in Hz)	HMBC Proton	COSY Proton	NOESY Proton
1	35.3, CH_2	2.18 (m)	H-2, H-25	H-2	H-25b, H-11a
		1.10 (m)		H-25a	
2	29.7, CH_2	2.13 (m)	H-1	H-1	
		1.67 (m)			
3	98.9, C	-	H-23, H-24, H-25b		
4	40.5, C	-	H-2, H-5, H-23, H-24, H-25		
5	49.9, CH	1.16 (m)	H-1, H-6, H-9, H-23, H-24	H-6, H-25b	H-23
6	19.9, CH_2	1.57 (m)	H-1, H-5, H-7, H-24	H-5, H-7	H-25a, H-7a
		1.45 (m)	H-26		
7	32.2, CH_2	1.43 (m)	H-5, H-6, H-26	H-6	H-26
		1.30 (m)			H-6b, H-27
8	39.9, C	-	H-9, H-26, H-27		
9	45.2, CH	1.42 (dd, 12.7, 2.7)	H-1, H-7, H-11, H-25, H-26	H-11	H-27
10	35.7, C	-	H-1, H-6, H-25		
11	22.4, CH_2	1.52 (m)	H-9, H-12, H-13	H-9, H-12	H-1a, H-23, H-25b, H-7b
		1.01 (m)			
12	25.6, CH_2	1.65 (m)	H-9, H-11, H-13, H-18	H-11	H-23
		1.02 (m)			
13	38.4, CH	1.66 (m)	H-15, H-18, H-19, H-27	H-12, H-18	H-19, H-26, H-28
14	42.7, C	-	H-13, H-15, H-26, H-27		
15	27.6, CH_2	1.61 (m)	H-16, H-27	H-16	H-26, H-28
		1.07 (m)			
16	35.6, CH_2	1.47 (m)	H-15, H-18, H-28	H-15	
		1.36 (m)			H-15b, H-27
17	43.2, C	-	H-15, H-16, H-22, H-28		
18	48.2, CH	1.40 (m)	H-16, H-19, H-28	H-13, H-19	H-27
19	48.0, CH	2.36 (m)	H-21, H-22, H-29, H-30	H-18, H-21	H-13, H-28
20	150.9, C	-	H-18, H-19, H-21, H-29, H-30		
21	30.0, CH_2	1.92 (m)	H-19, H-22, H-29, H-30	H-19, H-22	H-19, H-28
		1.31 (m)			
22	40.1, CH_2	1.38 (m)	H-28	H-21	H-28
		1.19 (m)			
23	27.0, CH_3	1.01 (s)	H-5, H-24		H-5, H-25b
24	18.5, CH_3	0.96 (s)	H-5, H-23		H-25a, H-6a
25	68.2, CH_2	4.24 (dd, 8.8, 2.9)	H-1, H-5, H-9, H-26	H-1b	H-1b, H-2a, H-6a, H-24, H-26
		3.72 (dd, 8.8, 1.9)		H-5	H-1a, H-11a, H-23, H-26
26	16.4, CH_3	0.94 (s)	H-7, H-9, H-15		H-25a, H-25b
27	14.4, CH_3	0.92 (s)	H-15		H-18
28	18.2, CH_3	0.78 (s)	H-16, H-18		H-13, H-19, H-21a, H-22a
29	109.5, CH_2	4.68 (d, 2.4)	H-19, H-30	H-30	H-30
		4.56 (dq, 2.7, 1.4)			H-30
30	19.5, CH_3	1.67 (s)	H-19, H-29	H-29	H-29

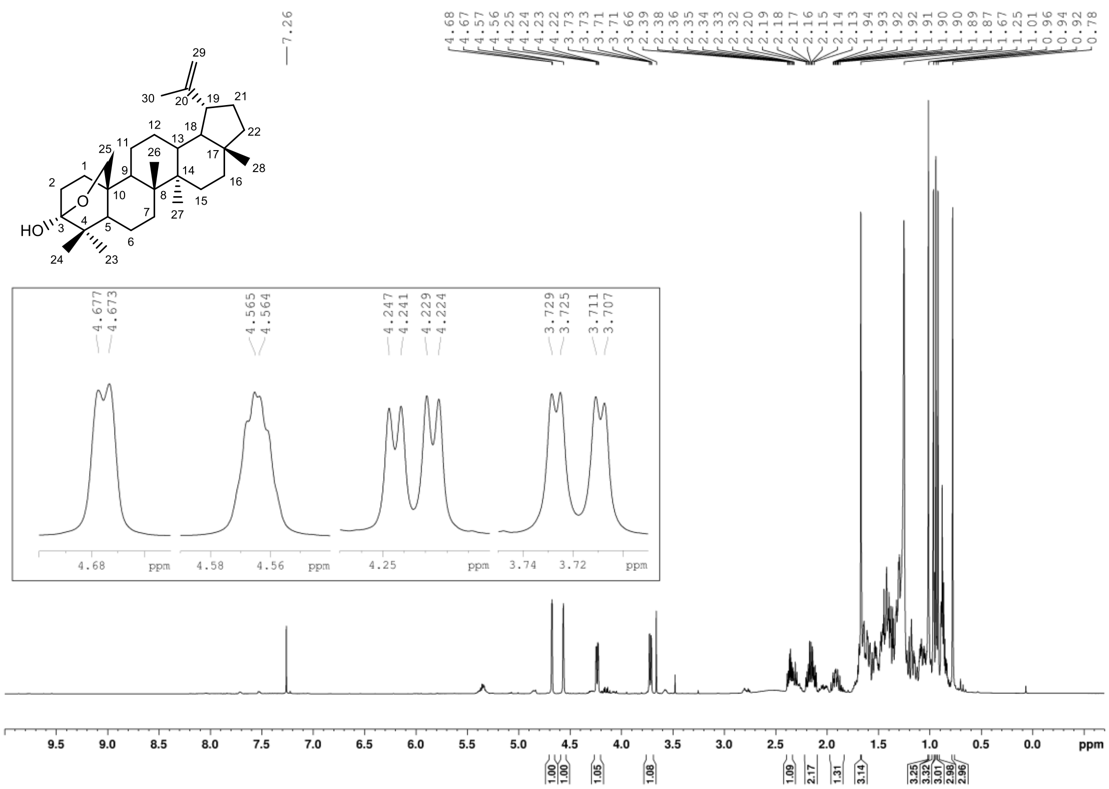

Figure S3. ^1H NMR spectrum ($CDCl_3$, 500 MHz) of compound **1.**

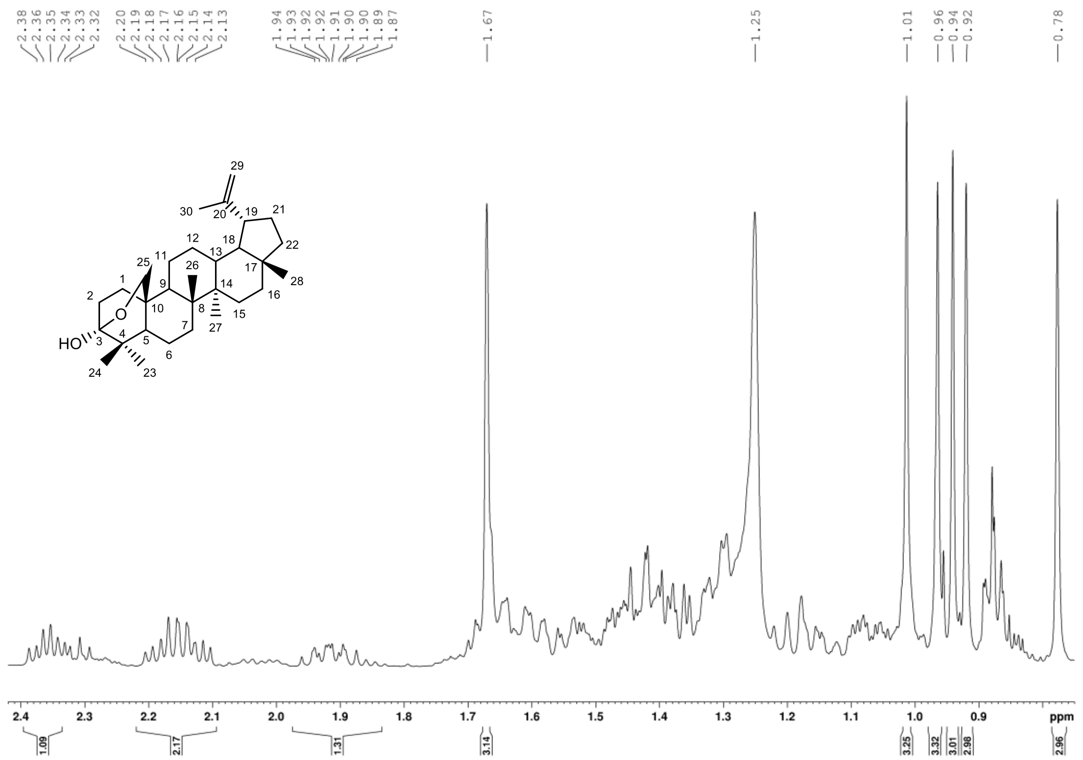

Figure S4. Enlarged view of ^{1}H NMR spectrum ($CDCl_3$, 500 MHz) of compound **1.**

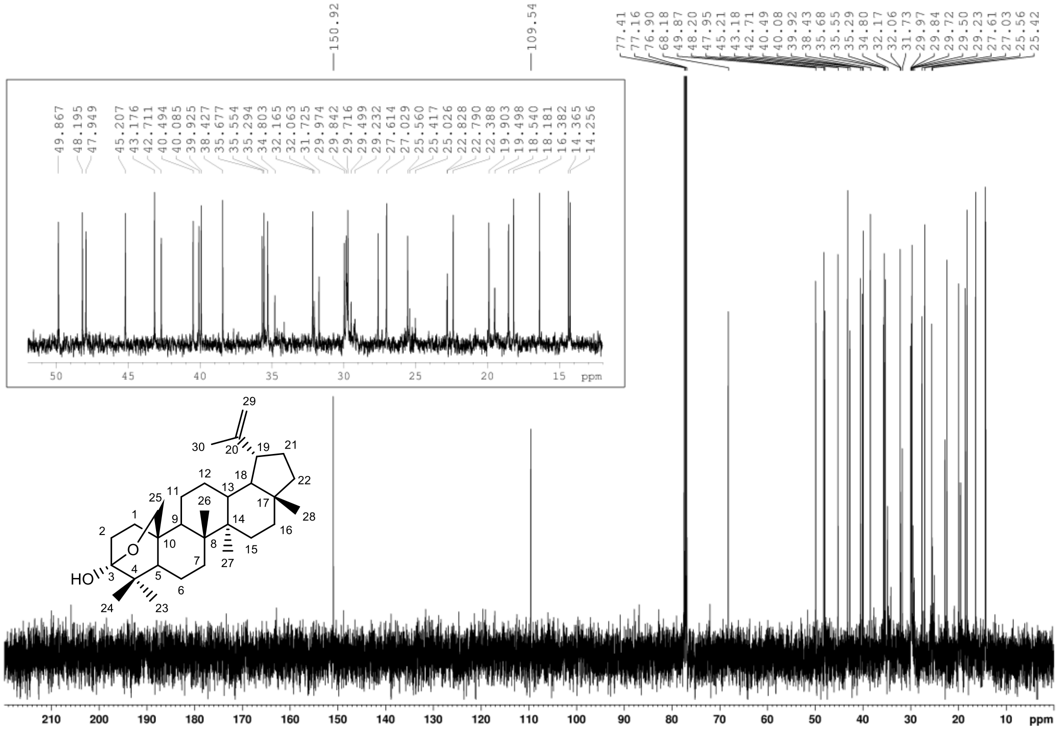

Figure S5. ^{13}C NMR spectrum ($CDCl_3$, 126 MHz) of compound **1.**

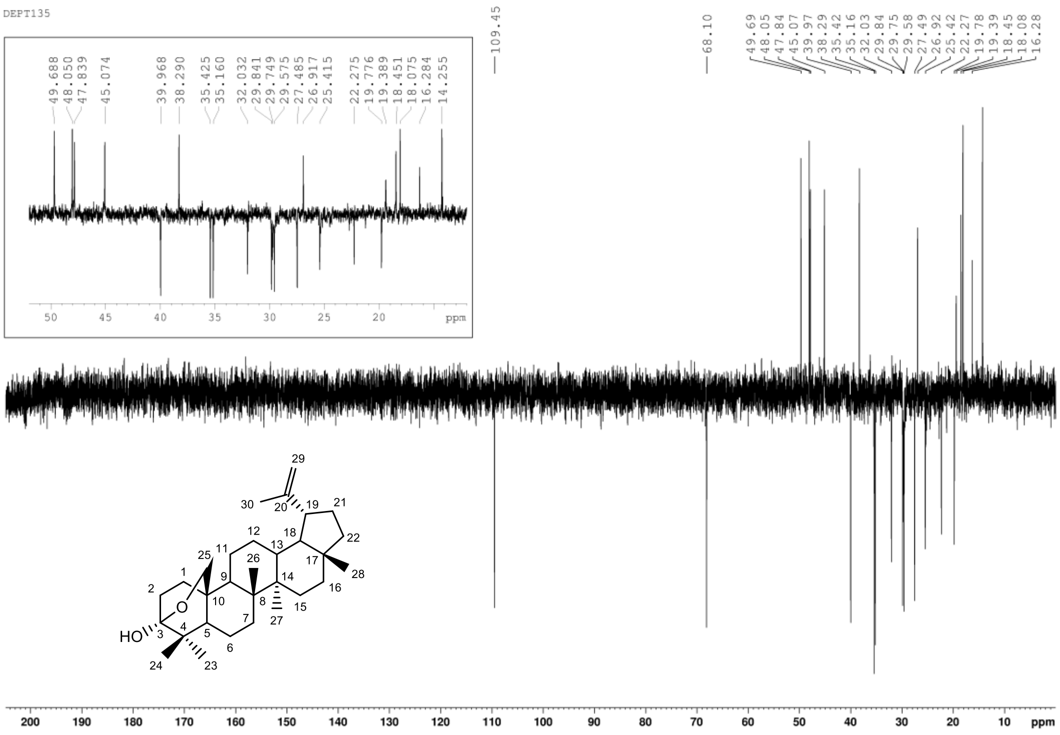

Figure S6. ^{13}C DEPT 135 NMR spectrum ($CDCl_3$, 126 MHz) of compound **1.**

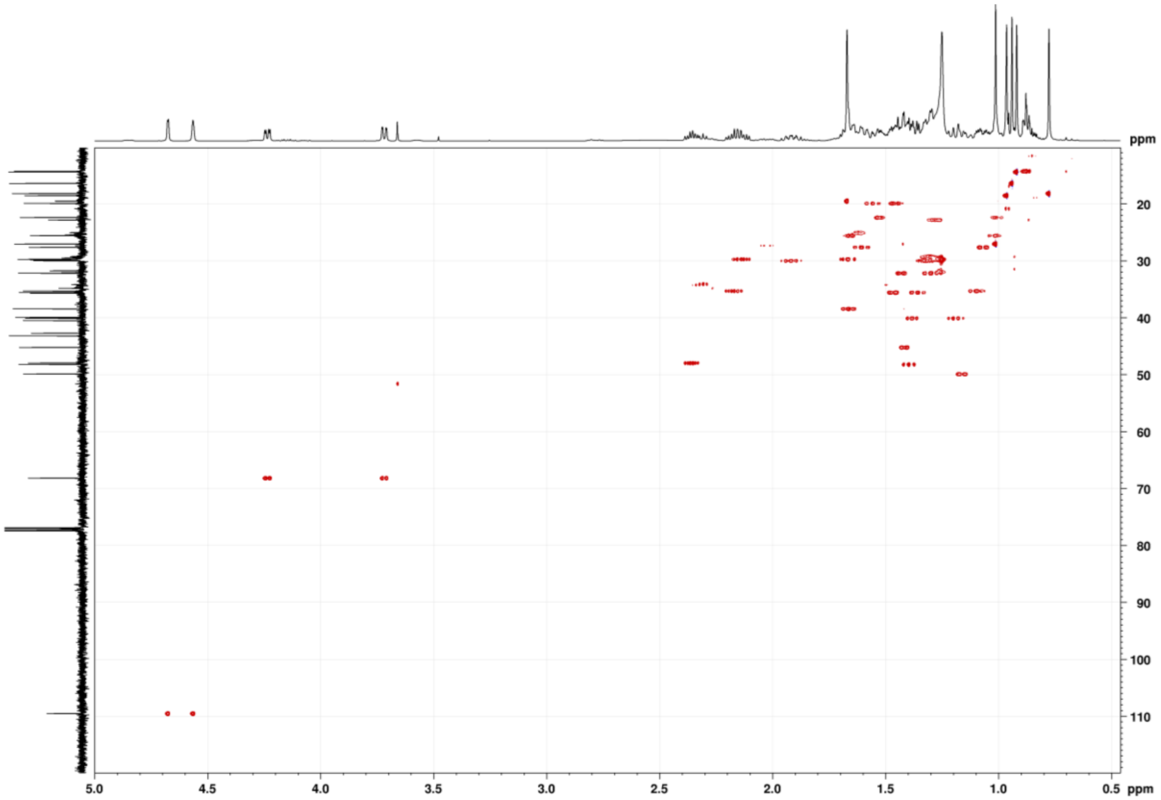

Figure S7. ^{1}H–^{13}C HSQC NMR spectrum ($CDCl_3$, 500 MHz) of compound **1.**

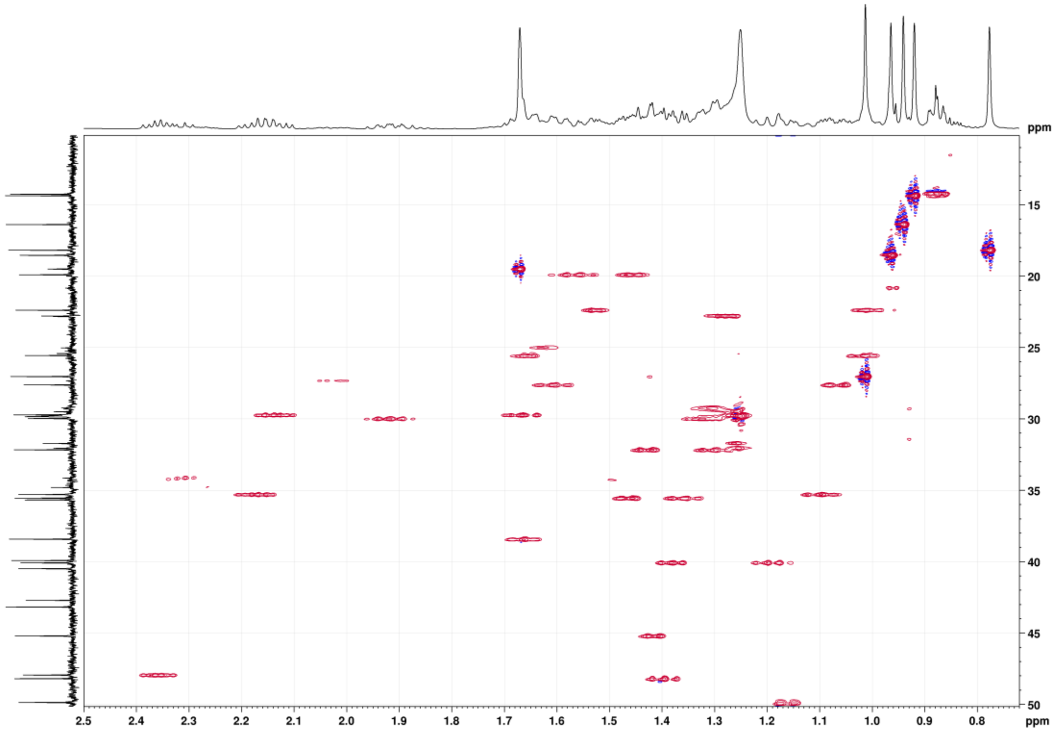

Figure S8. Band-selective ^{1}H–^{13}C HSQC NMR spectrum ($CDCl_3$, 500 MHz) of compound **1.**

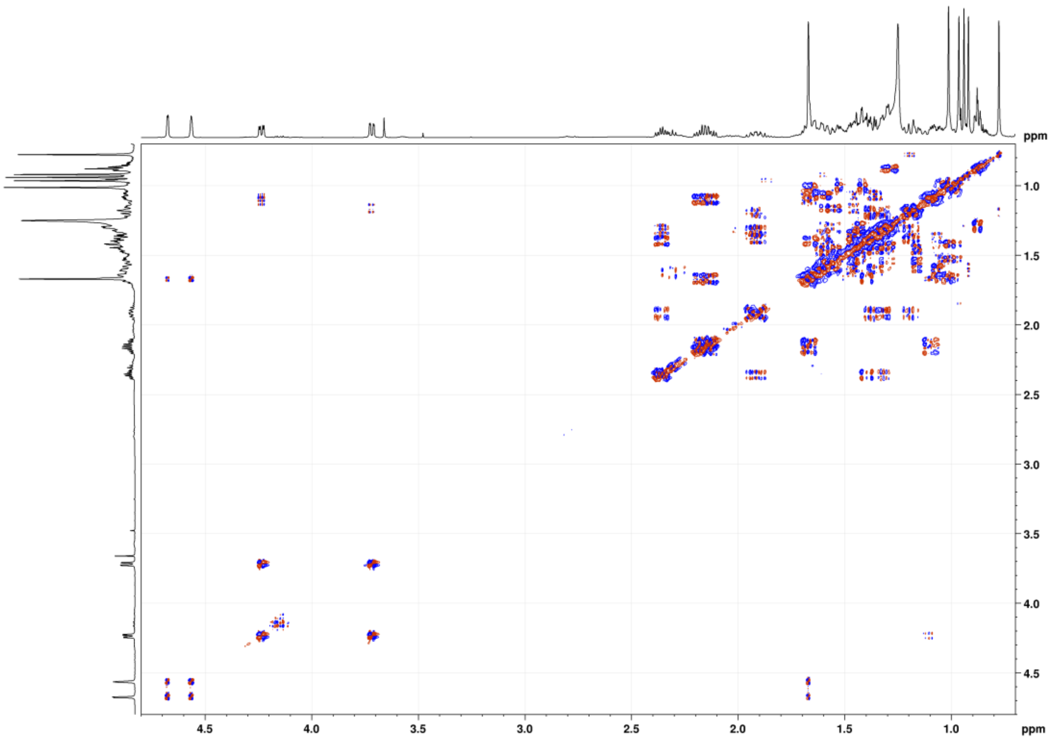

Figure S9. ^{1}H–^{1}H DQF COSY NMR spectrum ($CDCl_3$, 500 MHz) of compound **1.**

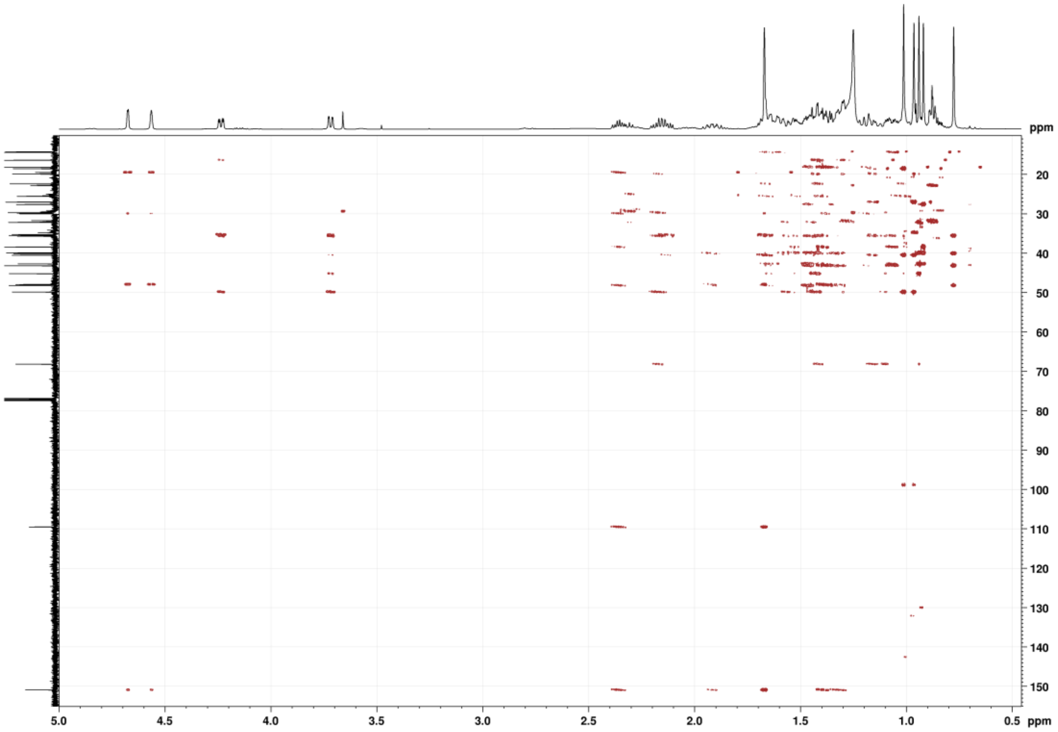

Figure S10. ^{1}H–^{13}C HMBC NMR spectrum ($CDCl_3$, 500 MHz) of compound **1.**

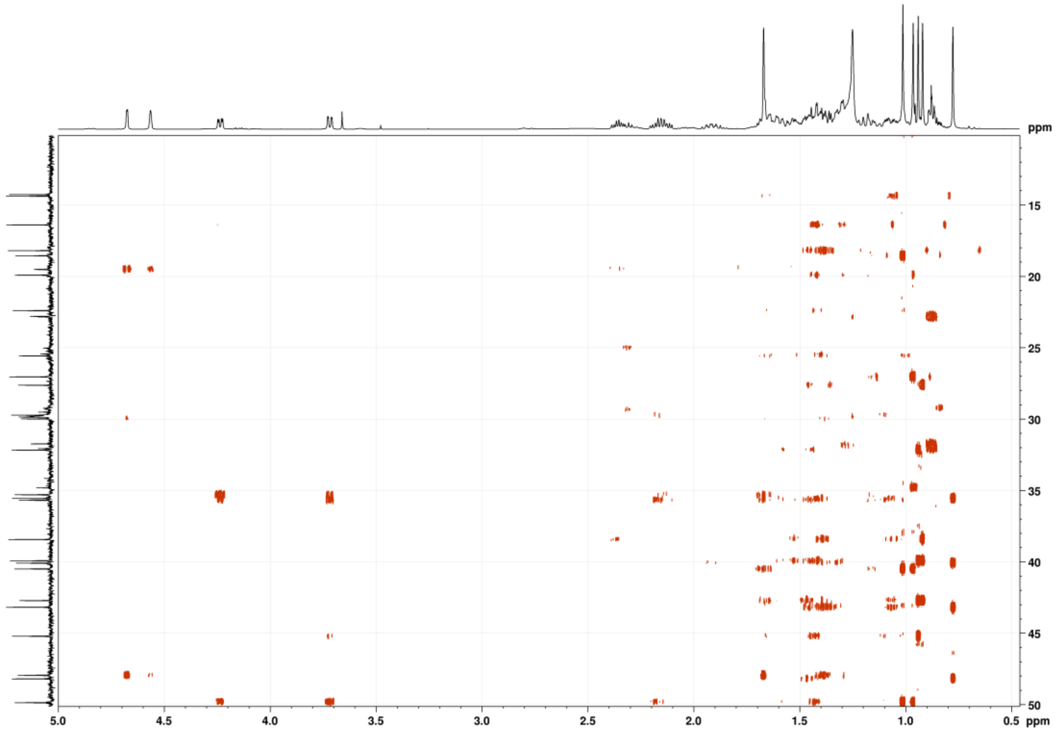

Figure S11. Band-selective ^{1}H–^{13}C HMBC NMR spectrum ($CDCl_3$, 500 MHz) of compound **1.**

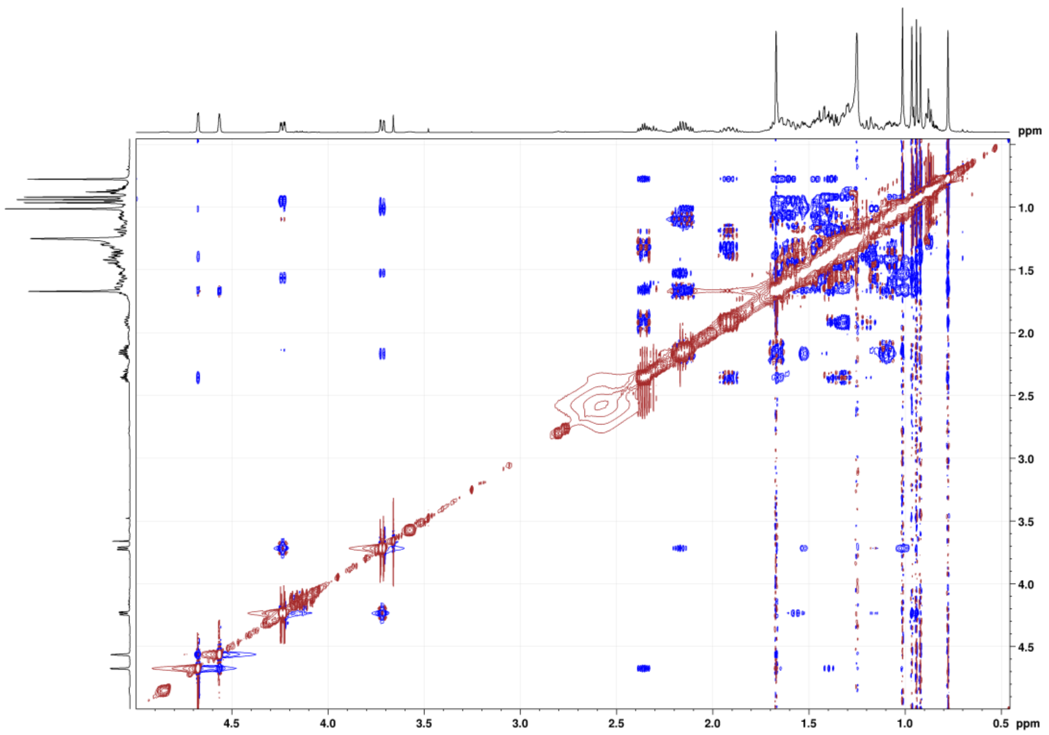

Figure S12. ^{1}H–^{1}H NOESY NMR spectrum ($CDCl_3$, 500 MHz) of compound **1.**

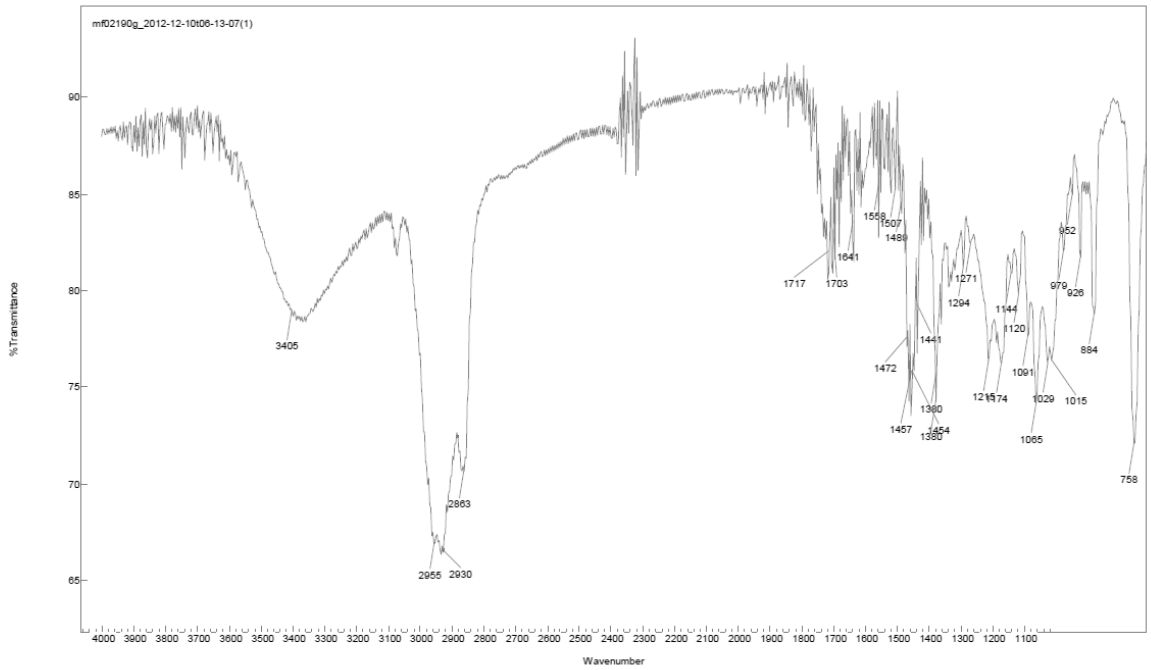

Figure S13. IR spectrum of compound **1.**
